# Why teachers do (or do not) implement recommended teaching practices? An application of the theory of planned behavior

**DOI:** 10.3389/fpsyg.2024.1269954

**Published:** 2024-03-13

**Authors:** Christophe Dierendonck, Débora Poncelet, Mélanie Tinnes-Vigne

**Affiliations:** Department of Education and Social Work, Institute for Lifelong Learning and Guidance, University of Luxembourg, Luxembourg, Luxembourg

**Keywords:** theory of planned behavior, teaching practices, competency-based practices, differentiated instruction, formative assessment, confirmatory factor analysis, structural equation modeling

## Abstract

**Introduction:**

In Luxembourg, competency-based practices (CBP), differentiated instruction (DI), and formative assessment (FA) have been imposed by the 2009 school law. Referring to the Theory of Planned Behavior (TPB), this study examined factors influencing the implementation of these practices in classrooms.

**Methods:**

Teachers participated in an online survey assessing their attitudes, subjective norm, perception of behavioral control, intention, and pedagogical practices regarding CBP, DI, or FA. Measurement models were used in structural equation models testing the TPB.

**Results:**

If the main relationships postulated by the theory were confirmed, some inconstancies were observed depending on the targeted practices. Structural equation TPB models controlling for gender, experience, teaching level, and socio-economic level of the school population explained between 20 and 45% of the variance in teachers’ practices, and between 65 and 75% of the variance in teachers’ intention to use these practices.

**Discussion:**

The relevance of the TPB for studying teaching practices and implications for professional training are discussed.

## Introduction

In the last three decades, globalization, international organizations (e.g., UNESCO), but also specific national contingencies have prompted many countries or regions (e.g., Canada, France) to reform their educational systems by releasing new curricula, by recommending new standards in terms of teaching, and by implementing new accountability policies (OECD, 2010, 2018; Maroy and Pons, 2021). In practice, the implementation of such policies has been difficult for many reasons such as limited budget, timing, coherence, communication, interactions between multiple stakeholders, lack of resources, support and expertise, lack of data and monitoring, lack of empirical findings, resistance to change as well as contextual factors (Fullan, 1991 2005 McLaughlin and Ruby, 2021). Nevertheless, effective reform implementation in the field of teaching practices depends mainly on teachers, and altering traditional teaching practices is a multifaceted undertaking influenced by numerous personal, organizational, social, and contextual factors (Thurlings et al., 2015).

In Luxembourgish elementary schools,^1^ competency-based practices (CBP), differentiated instruction (DI), and formative assessment (FA) have been mandatory since the reform of the education system in 2009. In this specific context, however, no study to date has examined either the actual implementation of the reform in the classrooms, nor the factors that lead teachers to adopt (or not) these recommended practices.

Based on teachers’ self-reports, the current study examines in what extent CBP, DI, and FA are implemented in elementary classrooms and highlights some determinants of these pedagogical practices through the lens of the Theory of Planned Behavior (TPB) (Ajzen, 1991). While the TPB has been extensively employed in health, psychology, and social domains to elucidate human behaviors, there is a growing trend in its application for predicting educational practices (e.g., MacFarlane and Woolfson, 2013; de Leeuw et al., 2015). The present study contributes to both theoretical and practical knowledge, by enhancing our understanding of predictive factors within the TPB framework, offering insights into how attitudes, subjective norm, and perceived behavioral control interact in the context of implementing specific teaching practices. The study also provides practical insights for intervention programs in schools and for teachers’ professional development by identifying key determinants that can enhance the use of CBP/DI/FA practices.

### Competency-based practices

A competency-based approach to instruction has been developed in opposition to traditional instruction. This latter approach struggled to manage the growing heterogeneity of students, both in terms of their academic levels and their learning pace. In traditional settings, time is considered as a constant and learning as a variable. In CBP, learning is the constant and time is the variable. This premise justified the creation of learning cycles in several European countries’ educational systems (e.g., Belgium, Luxembourg), with the goal of giving more time to pupils with a slower learning pace. According to Le et al. (2014), competency-based education has three main elements: mastery (pupils progress in their schooling after demonstration of their mastery of knowledge and skills, as defined by the competence curriculum, in which all pupils are set high learning targets), pacing (students’ progress at different learning rates in different areas, rather than pertaining to a teacher-driven, class-wide schedule), and instruction (students receive customized support which matches their individual learning needs). To give adequate support to students, a comprehensive assessment system—with formative and summative assessments—is an essential aspect (Pace, 2013). Formative assessments guide daily instruction and allow for the development of adequate support to address learning difficulties, while summative assessments are used to enable students to demonstrate mastery of skills they have developed during learning activities. In competency-based instruction, teachers are asked to develop higher-level skills (called competencies) through “learning situations” or “situational problems,” rather than teaching subject-based content through the previous objective-based approach (Jonnaert et al., 2007; Potvin et al., 2012). In other words, whereas the traditional approach gives prominence to skills at the lower levels of Bloom’s taxonomy (knowledge, understanding, application), the CBP place the focus on the higher levels of this taxonomy (analysis, synthesis, evaluation), involving skills of flexible thinking, problem solving and the application of knowledge in any context (Bingham et al., 2021). Exploring the antecedents and conditions of CBP are of capital importance. This will enable better understanding of which personal and contextual factors influence the use of this student-centered pedagogical approach in the classroom.

### Differentiated instruction

As suggested by Lafontaine (2017), school systems and teachers traditionally rely on two types of differentiation practices to address the heterogeneity of students: structural differentiation and differentiated instruction. Structural differentiation is external to the classroom. It refers to the organizational devices of the educational system and/or the school that make it possible to form learning groups more homogeneous in a permanent way (learning cycles, retention or lengthening of cycle, orientation toward a more or less demanding or prestigious school track or option, constitution of ability classes, …) or in a transitory way (additional remediation groups for students in difficulty, advanced courses for the best students, integration classrooms, …). Differentiated instruction is internal to the class. It refers to all teaching practices that aim to organize student learning by considering their individual differences (abilities, needs, etc.), while pursuing ambitious objectives common to all.

According to Tomlinson et al. (2003), differentiated instruction can be defined as “an approach to teaching in which teachers proactively modify curricula, teaching methods, resources, learning activities, and student products to address the diverse needs of individual students and small groups of students to maximize the learning opportunity for each student in a classroom” (p. 121). For Tomlinson and Imbeau (2010), “at the core of the classroom practice of differentiation is the modification of four curriculum-related elements—content, process, product, and affect—which are based on three categories of student needs and variances—readiness, interest, and learning profile” (p. 15). Roy et al. (2013) defined differentiated instruction as “an approach by which teaching is varied and adapted to match students’ abilities using systematic procedures for academic progress monitoring and data-based decision-making” (p. 1187). They added that their definition differs from the one proposed by Tomlinson et al. (2003) in three ways: “First, although individual differences may manifest themselves in more than one dimension, such as interests and learning profiles (Tomlinson et al., 2003), we only focus on differences in ability (in French and Math classes), which constitute the most important challenge in regular classrooms. Second, although it can be useful to distinguish between content, process, and product, we propose that all strategies aimed at varying instruction could be grouped under the concept of instructional adaptations. Third, we put emphasis on academic progress monitoring as it represents a distinct component of differentiated instruction” (p. 1187).

Although DI is a required pedagogical approach in many countries, only few studies have explored how DI practices are related to contextual or personal factors. These studies assume that DI implementation would be significantly associated with teachers’ DI self-efficacy (Dixon et al., 2014; Suprayogi et al., 2017; Whitley et al., 2019), teachers’ knowledge of DI (King et al., 2010), teachers’ beliefs (Santangelo and Tomlinson, 2012; Suprayogi et al., 2017; Whitley et al., 2019; Pozas et al., 2020), professional development (Dixon et al., 2014; Prast et al., 2018), organizational support (Whitley et al., 2019), school culture (Smit and Humpert, 2012), and classroom size (Tomlinson et al., 2003; Suprayogi et al., 2017).

### Formative assessment

Although the concept of FA has been defined in different ways in the literature (for a review, see Wiliam, 2018), it mainly refers to the idea that assessment information could be used to give feedback to students about their current learning process, and to teachers about the efficacy of the practices they used. Formative assessment thus involves collecting evidence about students’ learning and using this information to guide the students in their current and future learning (Schildkamp et al., 2020).

The positive impact of formative assessment on students’ achievement has been demonstrated for many years. Three relatively recent meta-analyses (Graham et al., 2015; Klute et al., 2017; Lee et al., 2020) quantified this impact. Average effect sizes ranged from *d* = 0.26 to *d* = 0.72, suggesting that formative assessment is somehow positively impacting students’ achievement. Based on its positive impact, formative assessment has been considered as an effective teacher practice and recommended in many educational systems. However, the implementation of formative assessment in teachers’ daily practice remains a substantial challenge because teachers often adopt only a few of the principles associated with formative assessment (goal setting, gathering data about students’ learning, giving feedback to students). Moreover, they do not fully integrate it into their teaching approach (Hunt and Pellegrino, 2002; Bennett, 2011; Wylie and Lyon, 2015; Heitink et al., 2016; Yan and Brown, 2021; Yan et al., 2021; Lui and Andrade, 2022). Exploring the antecedents and conditions of formative assessment practices are thus of prime importance when seeking to better understand which personal and contextual factors enable and maximize the use of formative assessment in the classroom (Heitink et al., 2016; Schildkamp et al., 2020; Yan et al., 2022).

### Theory of planned behavior

**Figure 1:**
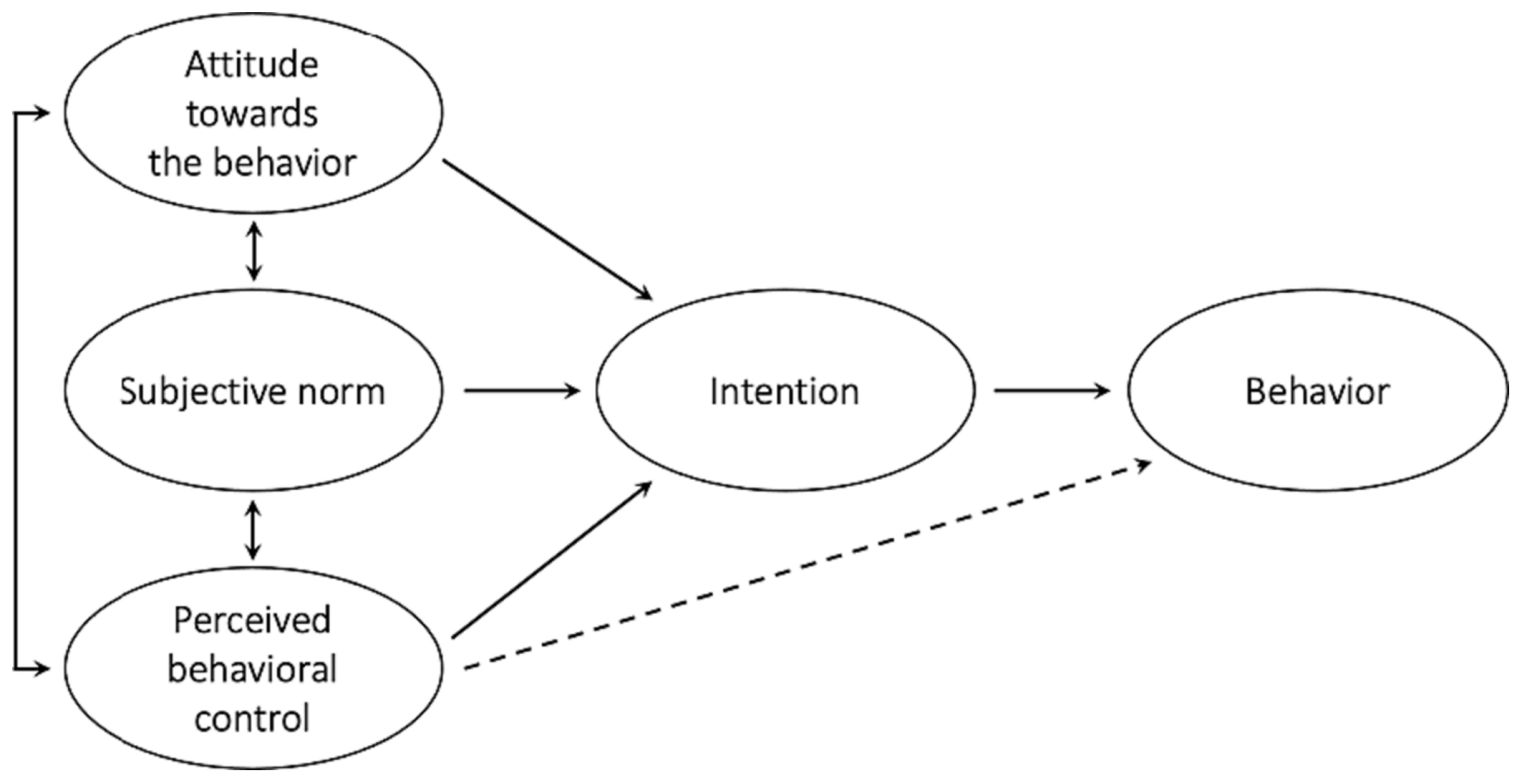
Theory of planned behavior (Ajzen, 1991).

The TPB has been used successfully in many fields to predict and explain specific behaviors (for a review, see Armitage and Conner, 2001). The most used version of the TPB model (Ajzen, 1991) is depicted in Figure 1. Behavior adoption is explained as a cognitive and emotional process consisting of four elements: (1) attitude toward the target behavior, (2) subjective norm related to the behavior, (3) perceived behavioral control, and (4) intention (or motivation) to adopt the target behavior. In the terms set out by Ajzen (1991), attitude taken toward the behavior refers to the degree to which a person has a favorable or unfavorable evaluation or appraisal of the behavior in question; subjective norm refers to the perceived social pressure to perform (or not) the behavior; perceived behavioral control refers to the perceived ease or difficulty of performing the behavior; and intention captures the motivational factors that influence a behavior. According to the theory, the intention to perform the target behavior is directly predicted by attitude, subjective norm, and perceived behavioral control, whereas the performance of the behavior is directly determined by intention and perceived behavioral control. Ajzen’s theory suggests the general rule that the more favorable the attitude and subjective norm with respect to a behavior, and the greater the perceived behavioral control, the stronger should be an individual’s intention to perform the behavior under consideration (Ajzen, 1991, p. 188). Moreover, the stronger the intention to engage in a behavior, the more likely should be its performance (Ajzen, 1991, p. 182).

**Figure 2:**
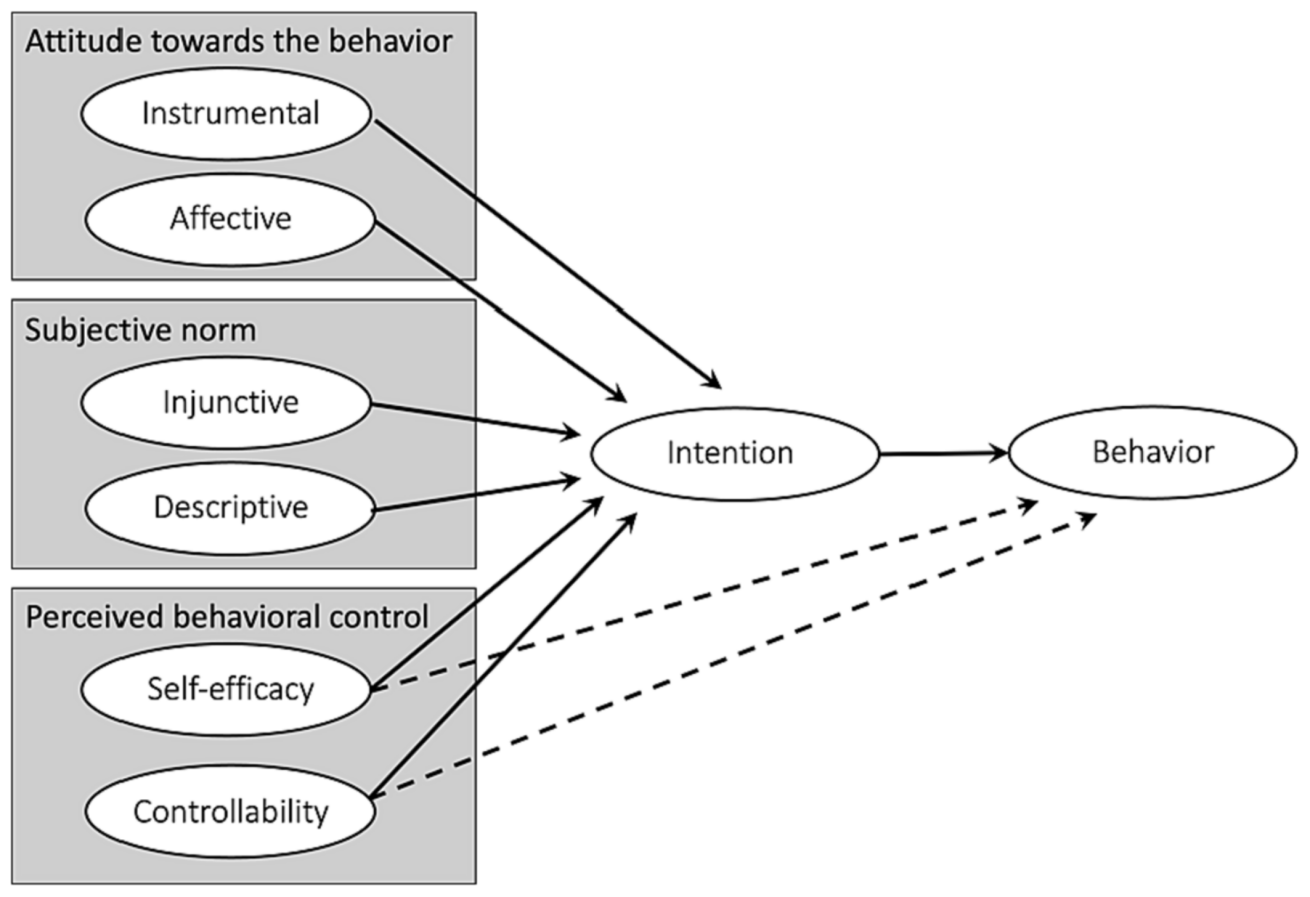
Illustration of a full two-component version of the TPB.

Reconceptualizations of the original model led to distinctions being made between instrumental attitude and affective attitude (e.g., Voet and De Wever, 2020), between injunctive norm and descriptive norm (e.g., Rivis and Sheeran, 2003 Knauder and Koschmieder, 2019), between self-efficacy and controllability (e.g., Ajzen, 2002), or to make two (e.g., Yan and Cheng, 2015) or three of these distinctions (e.g., Wilson et al., 2016) to increase the predictive strength of the TPB. Attitudes toward the target behavior may be instrumental (value/efficacy associated with the behavior, anticipated consequences if the behavior is performed) or affective (feelings or emotions arising from the idea of performing the behavior). Subjective norm regarding the target behavior may be injunctive (expectations of supervisors or reference groups regarding the adoption of the behavior) or descriptive (adoption or non-adoption of the behavior by others around the person). Perceived behavioral control may refer to the sense of efficacy or ability to perform the target behavior, or to the sense of autonomy in choosing to perform the behavior. In their study seeking to explain teachers’ reported inclusive behaviors, Wilson et al. (2016) illustrated the full two-component model of TPB, as depicted in Figure 2.

### A limited number of TPB studies regarding CBP, DI, and FA

TPB has been identified as a suitable framework to develop broad understanding of factors impacting inclusive teaching practices (e.g., Opoku et al., 2021), the latter being defined in a narrow sense referring to the inclusion of pupils with disabilities in mainstream schools (Sharma et al., 2017) or in a broader one referring to a complex combination of several pedagogical approaches such as teachers’ collaboration, feedback to student, individualized support, or grouping (e.g., Loreman, 2017).

While several studies have attempted to apply part of the TPB to teaching practices (e.g., Kupers et al., 2023; Urton et al., 2023), only few have tested the full model in relation with CBP, DI, or FA practices. Table 1 describes the only TPB studies we found concerning these teaching practices. As shown in the table, the TPB and the measures taken are operationalized in very different ways. Findings of these studies were also relatively contrasted.

**Table 1:** Operationalization of the mentioned TPB studies.

Study	Topic	TPB version	TPB predictors
Lenski et al. (2019)	Competency-based instruction	Full one-component	Attitude (6 items) Subjective norm (4 items) Perceived behavioral control (4 items) Intention (4 items)
Hellmich et al. (2019)	Everyday practices in inclusion classrooms	Full one-component	Attitudes toward inclusion (5 items) Perceived school management’s expectations (4 items) Collective self-efficacy beliefs (8 items) Intention (5 items)
Knauder and Koschmieder (2019)	Individualized student support	Full one-component	Attitude toward individualized support (6 items) Subjective norm (3 items) Influence of the school (6 items) Perceived behavioral control (6 items) Intrinsic intention to support (4 items) Extrinsic intention to support (3 items)
Yan and Cheng (2015) Karaman and Sahin (2017)	Formative assessment	Two-component for attitude One-component for subjective norm Two-component for PBC	Affective attitude (7 items) Instrumental Attitude (10 items) Subjective norm (5 items) Controllability (4 items) Self-efficacy (6 items) Intention (6 items)

Lenski et al. (2019) conducted a quantitative study about the determinants of competency-based instruction of 1,660 German secondary school mathematics teachers, under the framework of the full one-component version of the TPB. Controlling for age, gender, and school type, their TPB predictive structural equation model (SEM) showed a good fit (χ^2^ = 910.30, df = 267, *p* < 0.001, CFI = 0.94, RMSEA = 0.04, SRMR = 0.04). All the regression paths of the model were statistically significant (*p* < 0.05). Intention was significantly associated with perceived behavioral control (*β* = 0.33, *p* < 0.001), attitude (*β* = 0.11, *p* < 0.05) and subjective norm (*β* = 0.08, *p* < 0.01). Competency-based instruction was significantly and directly predicted by intention (*β* = 0.45, *p* < 0.001) and perceived behavioral control (*β* = 0.24, *p* < 0.05). The model explained 20% of the variance in competency-based instruction and 17% of the variance in intention. To our knowledge, CBP in elementary school have never been examined under the framework of the TPB.

Two SEM studies (Hellmich et al., 2019; Knauder and Koschmieder, 2019) have used the TPB to explore the determinants of DI in elementary school. Importantly, the focus of these studies was not exactly DI, but everyday practices in heterogeneous classrooms (Hellmich et al., 2019) or individualized student support (Knauder and Koschmieder, 2019). In the study conducted by Hellmich et al. (2019) in Germany, five scales were administered to 290 primary school teachers to measure attitude toward inclusion, perceived school management’s expectations, collective self-efficacy beliefs, intention, and everyday practices in heterogeneous classrooms. Two SEM models were tested. The first model considered Ajzen’s original theory while two additional unexpected direct relations were added in the second model (between attitude and behavior, and between school management’s expectations and behavior). Authors asserted that fit indices for Model 1 (χ^2^ = 716.81, df = 316, *p* < 0.001, CFI = 0.88, RMSEA = 0.07) and Model 2 (χ^2^ = 708.54, df = 314, *p* < 0.001, CFI = 0.88, RMSEA = 0.07) were nearly acceptable. In Model 1, all the regression path of the model were statistically significant (*p* < 0.05), except between self-efficacy and behavior. Intention was significantly associated with self-efficacy (*β* = 0.19, *p* < 0.01), attitude (*β* = 0.31, *p* < 0.001) and school management’s expectations (*β* = 0.33, *p* < 0.001). Everyday practices in heterogeneous classrooms were significantly and directly predicted by intention (*β* = 0.54, *p* < 0.001), but not by self-efficacy (*β* = −0.10, *p* > 0.05). The model explained 28% of the variance in the behavior and 33% of the variance in behavioral intention. As their Model 2 explained only 29% (+1% compared to model 1) of the variance in the behavior and 31% (−2% compared to model 1) of the variance in behavioral intention, we do not describe these results here. Knauder and Koschmieder (2019) collected data from 488 primary teachers in Austria. Deviating somewhat from TPB, the authors considered two models: one with intrinsic intention (χ^2^ = 452.98, df = 174, *p* < 0.01, CFI = 0.95, RMSEA = 0.057; SRMR = 0.039) and one with extrinsic intention (χ^2^ = 407.37, df = 158, *p* < 0.01, CFI = 0.95, RMSEA = 0.058; SRMR = 0.049). Then, structural analysis was conducted for both measurement models. Fit indices for the intrinsic intention SEM (χ^2^ = 453.86, df = 177, *p* < 0.01, CFI = 0.95, RMSEA = 0.057; SRMR = 0.039) and for the extrinsic intention SEM (χ^2^ = 412.67, df = 158, *p* < 0.01, CFI = 0.95, RMSEA = 0.057; SRMR = 0.050) were considered as acceptable. In Model 1, intrinsic intention was only significantly explained by the attitude toward individualized support (*β* = 0.85, *p* < 0.01). Individualized support was significantly associated with intrinsic intention (*β* = 0.23, *p* < 0.01) and perceived behavioral control (*β* = 0.41, *p* < 0.001). The model explained 36% of the variance in the behavior and 77% of the variance in behavioral intention. In Model 2, extrinsic intention was only significantly explained by the school influence (*β* = 0.71, *p* < 0.01). Individualized support was only significantly associated with perceived behavioral control (*β* = 0.53, *p* < 0.01). All other paths were non-significant. The model explained 34% of the variance in the behavior and 64% of the variance in behavioral intention. The authors concluded that the TPB may not be able to capture the specificity of individualized support.

Yan and Cheng (2015) and Karaman and Sahin (2017) conducted the only two quantitative studies about the determinants of formative assessment under the framework of the TPB. Yan and Cheng (2015) developed the *Teachers’ Conceptions and Practices of Formative Assessment Scale* (TCPFS), a 40-item instrument based on an extended version of the TPB. To assess the construct validity of the TCPFS scales, the authors used Rasch analysis, arguing that there are weaknesses with conventional techniques such as factor analysis considering Likert-type scales as interval scale data.^2^ They concluded that most of their scales had quite good psychometric properties, while the subjective norm scale only showed acceptable quality. Nevertheless, by apparently conducting seven separate Rash analyses, Yan et al. were unable to verify that their scales were indeed independent. For example, affective attitude and instrumental attitude are considered as independent scales, but they appeared to be highly correlated (*r* = 0.82), suggesting that items of both scales have maybe more in common than expected. The Rasch-calibrated measures were included in a path analysis model which showed an excellent fit (χ^2^ = 7.678, df = 3, *p* > 0.05, CFI = 0.997, TLI = 0.971, RMSEA = 0.059). Intention was significantly associated with self-efficacy (*β* = 0.44, *p* < 0.01), instrumental attitude (*β* = 0.34, *p* < 0.01), and subjective norm (*β* = 0.14, *p* < 0.01). Formative practices were significantly and directly predicted only by intention (*β* = 0.18, *p* < 0.01). The standardized regression weight of the direct paths from controllability and self-efficacy to behavior were not statistically significant. Importantly, the model explained 51% of the variance in teachers’ intention but only accounted for 6% of the variance in teachers’ formative assessment practices. In their study involving 400 primary teachers, Karaman and Sahin (2017) adapted the TCPFS to Turkish culture and examined teachers’ intentions and behaviors regarding formative assessment through structural equation modeling. Unlike Yan et al.’s study, they used confirmatory factor analysis and Cronbach’s Alpha reliability coefficient to assess the construct validity of the Turkish version of the TCPFS measurement scales. Their final confirmatory factor model showed an acceptable fit (χ^2^ = 1906.27, df = 567, *p* < 0.01, CFI = 0.90, TLI = 0.87, RMSEA = 0.07), except for TLI. Contrary to the authors’ assertion, several fit indices of their predictive model did not reach acceptable cut-offs (χ^2^ = 2051.97, df = 607, *p* < 0.01, CFI = 0.88, TLI = 0.87, RMSEA = 0.07). Intention was significantly associated with self-efficacy (*β* = 0.55, *p* < 0.01), controllability (*β* = 0.16, *p* < 0.01) and instrumental attitude (*β* = 0.16, *p* < 0.01). Formative practices were significantly and directly predicted by controllability (*β* = −0.52, *p* < 0.01) and self-efficacy (*β* = 0.68, *p* < 0.01). Importantly, the standardized regression weight of the path from intention to behavior was negative, but not statistically significant (*β* = −0.17). The model explained 71% of the variance in teachers’ intention and 15% of the variance in teachers’ formative assessment practices.

### The present study

Some of the previous studies showed that the TPB could be an interesting theoretical framework to explain teachers’ use of specific practices, but findings of these studies are contrasted. The present study offers the opportunity to simultaneously examine three recommended teaching practices in elementary school through the lens of the same version of the TPB and with similar measurement scales. The aim of the study is to analyze the network of relationships that emerged between TPB variables and to identify potential determinants for the adoption of these teaching practices.

According to the TPB theory, the following hypotheses are tested for CBP/DI/FA practices:

1. The full two-component version of the TPB provides a significantly better fit to the data, and accounts for a higher proportion of behavior variance than the original one-component version of the TPB (Ajzen, 1991).
2. Teachers’ practices are directly predicted by intention and perceived behavioral control. In other words, CBP/DI/FA practices will be more frequent if intention and perceived behavioral control are high.
3. Teachers’ intention is directly predicted by attitude, subjective norm, and perceived behavioral control. In other words, more positive attitude, subjective norm, and higher perceived behavioral control will be associated with higher intention to adopt the target behavior.

## Methods

### Procedure and participants

The study was approved by the Ethics Review Panel of the University of Luxembourg. In November and December 2021, the 5,905 elementary school teachers in Luxembourg were asked to participate in a national consultation mandated by the Educational Quality Observatory and funded by the Ministry of Education. The consultation covered a wide range of subjects (work engagement, job satisfaction, daily difficulties, collaboration, school functioning, parental involvement, teaching and assessment practices, teachers’ pedagogical beliefs, …), but the first aim was to take stock of the reforms to the education system since 2009.

We elaborated a planned missing data design (Graham et al., 2006) to cover all topics and to reduce as much as possible the time needed to complete the survey. An extract from this planned missing data design is given in Supplementary Appendix 2. Six versions of the questionnaire were developed. So, there were common items in all versions and specific items in each version of the questionnaire. Versions 1, 2, and 3 of the questionnaire focused on CBP, DI, and FA, respectively. Except for self-efficacy and intention items which were proposed in the form of a cursor to be moved in order to vary the ways in which questions were asked, respondents had the possibility to give the answers “not applicable” (coded 777) or “I do not know” (coded 888) to each item. For all items except those related to self-efficacy and intention, it was not possible to skip the items without giving an answer. Missing data were considered as missing answer by design (coded 999), or true missing answer (coded −999) in the case of self-efficacy and intention items.

Questionnaires were assigned randomly (version 1 was assigned to 985 teachers and versions 2–6 were each assigned to 984 teachers). Each elementary school teacher received a personal e-mail invitation and a unique access code enabling to answer one of the six versions of the questionnaire.

Teachers were briefed on the nature of the questionnaire and on how the answers would be treated in confidence. Participation was free and anonymous. The survey was delivered through Qualtrics. Instead of the estimated 50–60 min, some teachers needed between 60 and 90 min to fully complete the questionnaire. As shown by log data, most of them completed part of the questionnaire on 1 day and the other part on another day, as suggested in the survey instructions.

Of the 1825 teachers who take part to the survey, only 1,000 (193 for version 1, 150 for version 2, 164 for version 3, 154 for version 4, 157 for version 5, and 182 for version 6) reached the end of their questionnaire, suggesting that even with the planned missing data design, the task was undoubtedly too demanding for many. The 825 respondents dropped off at various points in their questionnaire: 369 in the first quarter, 325 in the second quarter, 77 in the third quarter, and 54 in the fourth quarter. Table 2 describes the two samples of respondents.

**Table 2:** Description of the two samples of respondents.

	Sample A Participants who reached the end of their questionnaire (*N* = 1,000)	Sample B Participants who gave up before the end of their questionnaire (*N* = 825)
% women	80.0	81.9
Mean experience (in years)	14.8	12.4
% cycle 1	23.2	23.5
% cycle 2	25.5	25.9
% cycle 3	23.2	17.3
% cycle 4	19.2	24.7
% two cycles or more	8.9	8.6

In order to identify (and exclude) careless respondents (Meade and Craig, 2012; Ward and Meade, 2023) in sample A, we conducted several analyses. First, we examined the response time computed by the Qualtrics platform. As the respondents could take a break or complete their questionnaire in several times, we only focused on the identification of too quick respondents. We used the 2 s per items cut-off suggested by Bowling et al. (2016) to identify careless responding. Even with a 3 s per items cut-off, any respondent of sample A was identified as careless respondent. In a second analysis, we checked the invariability of responses (i.e., consecutive identical responses) by examining within-person variance for the six sets of attitudinal and behavioral items under study. Lastly, we considered respondents with more than 20 “*Not applicable*” answers as careless respondents. In total, we identified and excluded 48 careless respondents in sample A. The “*I do not know*” and “*Not applicable*” answers were further recoded as missing value. The final analytic sample (*N* = 952) is composed of 79.9% of women. The mean experience is 14.9 years and teachers are 22.8% in cycle 1, 25.7% in cycle 2, 23.8% in cycle 3, 19.5% in cycle 4, and 8.2% in more than one cycle.

### Measures

All items and their descriptive statistics are reported in Supplementary Appendices 3–5. Items were administered in French. Scale development followed the suggestions made by DeVellis (2012). We first generate a pool of items which was reviewed by a panel of three experts. We then organized two group sessions in which a few teachers answered the items before discussing them with the research team to ensure that no item posed a problem of comprehension or led to misinterpretation. The questionnaire was finally pretested with 88 teachers during a pilot-study in May 2021. The final version of the measurement scales was designed based on the results of this pilot-study. Some items were amended, and we tried to optimize the length of the measurement scales when possible.

*Attitude* regarding CBP/DI/FA was measured with 6 original items. Three items examined instrumental attitude (e.g., *CBP/DI/FA is an effective way to improve school learning*) and 3 items assessed affective attitude (e.g., *Practicing CBP/DI/FA is professionally satisfying*). All items were rated using a six-point scale (Totally disagree, Disagree, Rather disagree, Rather agree, Agree, Totally agree).

*Subjective norm* regarding CBP/DI/FA was assessed using 6 original items. Three items covered the injunctive facet (e.g., *The regional director encourages me to further develop my CBP/DI/FA practices*) while 3 items measured the descriptive facet of subjective norm (e.g., *In my school cycle, CBP/DI/FA is at the heart of teaching practice*). All items were rated using a six-point scale (Totally disagree, Disagree, Rather disagree, Rather agree, Agree, Totally agree).

*Perceived behavioral control* regarding CBP/DI/FA was assessed using 8 original items. Four items examined the teachers’ self-efficacy facet (e.g., *To what extent do you feel competent to practice CBP/DI/FA?*) and four items assessed the controllability facet (e.g., *It is up to me to develop my practices further in line with CBP/DI/FA*). Items related to self-efficacy were rated using a cursor to move between 0 and 6 (0 = Not competent at all, 6 = Extremely competent) and controllability items were rated using a six-point scale (Totally disagree, Disagree, Rather disagree, Rather agree, Agree, Totally agree).

*Intention* regarding CBP/DI/FA consisted of 3 items measuring the motivation of teachers to implement CBP/DI/FA (e.g., *To what extent are you determined to use CBP/DI/FA?*). Items were rated using a cursor to move between 0 and 6 (0 = Very little determined, 6 = Very determined).

*Practices* regarding CBP/DI/FA were measured with original items or existing scales. Teachers were asked to report the frequency of several practices using a seven-point scale (Never, Rarely, Sometimes, Regularly, Frequently, Very frequently, Systematically). CBP practices were measured with 5 original items (e. g. *I organize lessons/activities where students practice transferring their knowledge, skills, and attitudes to other situations.*). DI practices were measured through the instrument developed by Roy et al. (2013). Seven items assessed the instructional adaptations dimension (e.g., *I plan different assignments to match students’ abilities*) and 4 items assessed the progress monitoring dimension (e.g., *I analyze data about students’ academic progress*). Due to the significant correlation among the behavioral items related to Differentiated Instruction (DI), we opted to focus on a singular behavioral dimension. This dimension combined items associated with instructional adaptations and progress monitoring. FA practices were measured with 6 original items (e. g. *I observe pupils while they are conducting a particular task in class and give them direct feedback on their work*).

### Data analysis

We followed the two-step approach defined by Anderson and Gerbing (1988). In the first step, the measurement part of the model was tested using confirmatory factor analysis (CFA) to evaluate the extent to which the set of indicators or items measures the latent factors they are supposed to measure. Then, a full model combining the measurement part and the structural parts was fitted through structural equation modeling (SEM) and estimates of indirect effects and their standard errors were calculated. Lastly, four covariables (gender, experience, teaching level, and socio-economic level of the school population) were added to the SEM models.

To respect the ordinal level of measurement of Likert-type items, all analyses were conducted using the robust weighted least squares mean and variance adjusted (WLSMV) estimator as implemented in Mplus 8.3 (Muthén and Muthén, 1998-2017). With this estimator, all available information is used, and missing values are handled using pairwise present.

The fit of the models was assessed considering several indices (χ^2^ statistics, CFI, TLI, RMSEA, and SRMR) and typical interpretation guidelines (Hu and Bentler, 1999). If statistically significant, the χ^2^-value advocates for rejecting the null hypothesis of a good fit. Nevertheless, this statistic is highly sensitive to sample size, and its significance should not be the only reason to reject a model. To be considered as acceptable, CFI and TLI must be equal or above 0.90 and RMSEA and SRMR must be less than 0.08. Scale reliability was estimated based on the CFA model results as suggested by Bollen (1989) and Wang and Wang (2020).

## Results

### Descriptive statistics

Descriptive results globally indicate that teachers tend to report a positive instrumental attitude and a positive affective attitude regarding CBP/DI/FA practices. For example, 72, 95, and 84% of the respondents, respectively, agreed with the item “*CBP/DI/FA practices are an effective way to improve school learning*,” and 88, 87, and 88% of the respondents, respectively, disagreed with the item “*CBP/DI/FA practices are demotivating*.”

A significant number of teachers feel a certain pressure to further implement CBP/DI/FA practices due to the injunctive norm or the descriptive norm. For example, 38, 49, and 36% of the respondents, respectively, agreed with the item “*I feel a certain amount of social pressure (from regional direction, colleagues and/or parents) to further develop my CBP/DI/FA practices*,” and 82, 75, and 76% of the respondents, respectively, agreed with the item “*In my cycle, CBP/DI/FA is at the heart of teaching practices*.” Concerning controllability and self-efficacy, teachers are also mostly positive. Intention to use CBP/DI/FA practices is very high as 80, 90, and 80% of the teachers are at least “*somewhat determined*” to implement target practices, respectively. Concerning the frequency of CBP/DI/FA practices, around 60%, 65–70%, and 60–70% of the teachers reported a frequent use, respectively.

### Measurement models

The first research question consisted in examining which of the one-component model or the two-component model was the most relevant TPB modeling regarding CBP/DI/FA. Fit indices of the rival CFA measurement models tested are provided in Table 3.

**Table 3:** Fit of CFA measurement models regarding CBP/DI/FA.

			Chi-square	df	CFI	TLI	RMSEA [90% CI]	SRMR
Full two-component models	2-2-2	M1-CBP	1088.052*	322	0.938	0.927	0.050 [0.047; 0.053]	0.087
		M2-DI	1167.989*	532	0.977	0.974	0.035 [0.033; 0.038]	0.072
		M3-FA	974.673*	349	0.939	0.930	0.043 [0.040; 0.047]	0.088
Hybrid models	1-2-2	M4-CBP	736.144*	254	0.955	0.947	0.045 [0.041; 0.048]	0.069	M5-DI	961.296*	443	0.980	0.978	0.035 [0.032; 0.038]	0.066
		M6-FA	661.079*	278	0.955	0.947	0.038 [0.034; 0.042]	0.068
1-1-2		M7-CBP	753.951*	260	0.954	0.947	0.045 [0.041; 0.048]	0.072
	M8-DI	1016.573*	449	0.978	0.976	0.036 [0.033; 0.039]	0.066
	M9-FA	635.792*	284	0.959	0.953	0.036 [0.032; 0.040]	0.069
Full one-component models	1-1-1	M10-CBP	902.221*	265	0.941	0.933	0.050 [0.047; 0.054]	0.082
		M11-DI	1178.712*	454	0.972	0.970	0.041 [0.038; 0.044]	0.070
		M12-FA	847.044*	289	0.934	0.926	0.045 [0.042; 0.049]	0.083

*p ≤ 0.001. df, degrees of freedom; CFI, Comparative Fit Index; TLI, Tucker-Lewis Index; RMSEA, Root Mean Square Error of Approximation; CI, Confidence Interval; SRMR, Standardized Root Mean Squared Residual.

We began by testing the full two-component models regarding CBP/DI/FA. Fit indices for the three two-component models (M1, M2, M3) were acceptable to good, but standardized correlations above 0.90 were observed between the two attitudinal scales, suggesting that both scales measured in fact the same latent factor. The MPlus software additionally highlighted some problems of negative residual variance or non-significant estimate for three items (N3: *I feel a certain amount of social pressure (from regional direction, colleagues and/or parents) to further develop my CBP/DI/FA practices*; N4: *In my cycle, CBP/DI/FA is at the heart of teaching practices*; C2: *I decide for myself whether or not to develop my practices further in line with CBP/DI/FA*). Models M4, M5, and M6 without the three previous items and with one component for attitude, two components for subjective norm, and two components for PBC were then tested. Fit indices were all excellent, but M5 showed a correlation greater than one between the two latent variables measuring injunctive and descriptive norms. In models M7, M8, and M9, we considered one component for attitude, one component for subjective norm, and two components for PBC. Fit indices for these models were good to excellent. We finally examined the full one-component models (M10, M11, M12) regarding CBP/DI/FA. Fit indices for these models were again good to excellent.

For all hybrid models (excepted M5) and full one-component models, we calculated a coefficient of reliability for each measurement scale (Table 4). All reliability coefficients were above 0.70. Moreover, all items loaded significantly and substantially on their hypothesized dimension. As several models showed a good fit to the data, and in order to respect the principle of parsimony, we retained the full one-component models as final measurement models. Items standardized estimates for these final models are reported in Supplementary Appendices 3–5.

**Table 4:** Scale reliability depending on CFA model.

	Hybrid models						Full one-component models		
	M4-CBP	M5-DI	M6-FA	M7-CBP	M8-DI	M9-FA	M10-CBP	M11-DI	M12-FA
Attitude	0.896	–	0.871	0.896	0.916	0.871	0.904	0.916	0.877
Subjective norm	–	–	–	0.807	0.711	0.795	0.806	0.710	0.795
Injunctive	0.805	–	0.747	–	–	–	–	–	–
Descriptive	0.802	–	0.801	–	–	–	–	–	–
PBC	–	–	–	–	–	–	0.848	0.869	0.854
Controllability	0.761	–	0.797	0.762	0.760	0.798	–	–	–
Self-efficacy	0.939	–	0.937	0.939	0.939	0.937	–	–	–
Intention	0.842	–	0.834	0.843	0.910	0.834	0.839	0.909	0.830
Behavior	0.884	–	0.795	0.884	0.941	0.795	0.890	0.941	0.806

### SEM models

For CBP, DI, and FA, the results of the structural equation modeling (including the measurement part and the structural part) showed a satisfactory fit, as shown in Table 5.

**Table 5:** Fit of SEM models regarding CBP/DI/FA.

SEM models	Chi-square	df	CFI	TLI	RMSEA [90% CI]	SRMR
CBP	915.721*	267	0.940	0.932	0.051 [0.047; 0.054]	0.085
DI	1195.356*	456	0.972	0.969	0.041 [0.038; 0.044]	0.071
FA	840.694*	291	0.935	0.928	0.045 [0.041; 0.048]	0.084

*p ≤ 0.001. df, degrees of freedom; CFI, Comparative Fit Index; TLI, Tucker-Lewis Index; RMSEA, Root Mean Square Error of Approximation; CI, Confidence Interval; SRMR, Standardized Root Mean Squared Residual.

**Figure 3:**
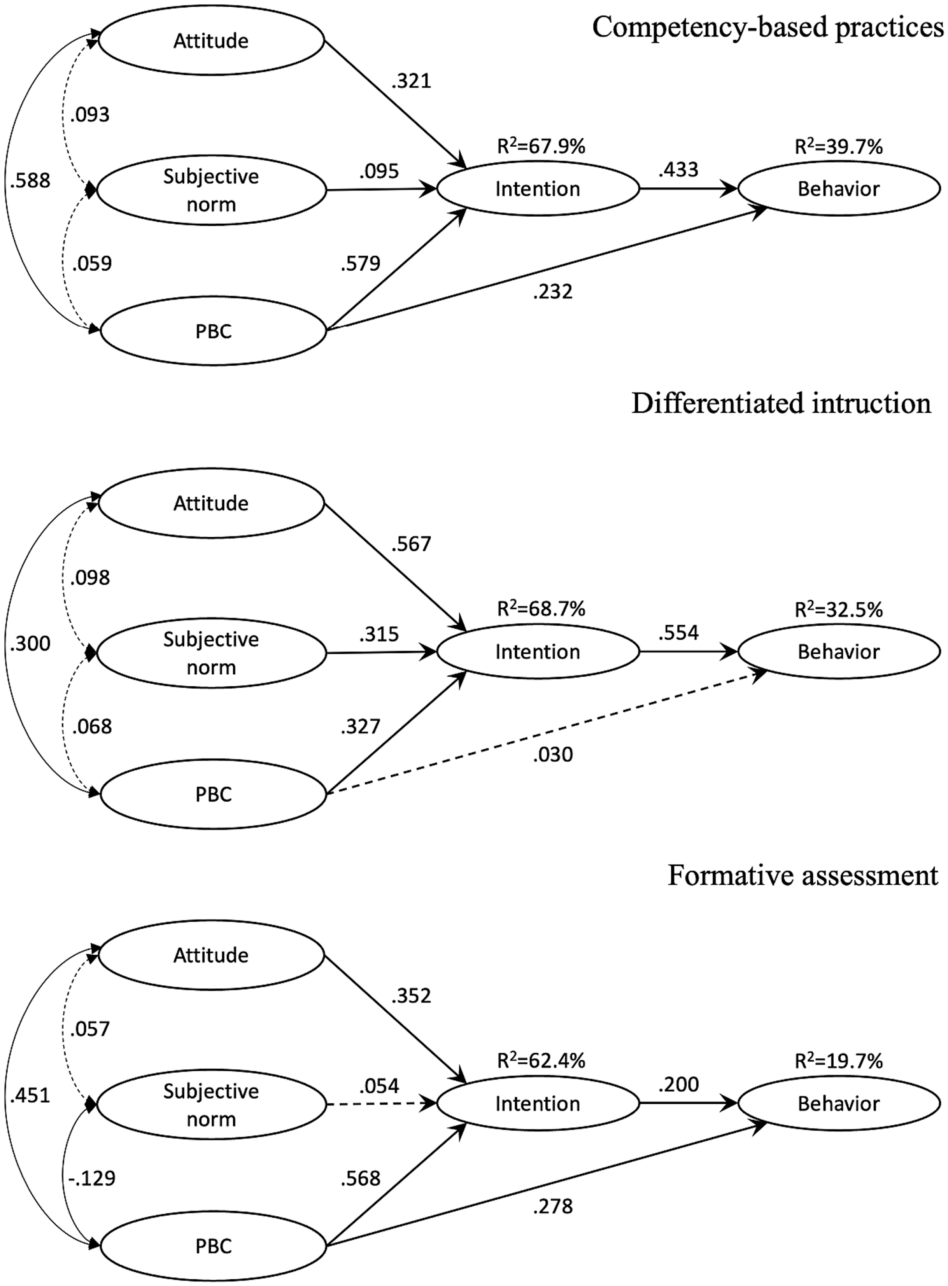
TPB models regarding CBP, DI, and FA.

Relationships between variables are depicted in Figure 3, for CBP/DI/FA. The measurement parts of the models are not presented for clarity purpose, and relationships represented by dotted lines are not statistically significant.

Concerning CBP, the standardized regression paths from intention to behavior (*β* = 0.433, *p* < 0.001) and from PBC to behavior (*β* = 0.232, *p* < 0.001) were statistically significant. The significant predictors of intention were attitude (*β* = 0.321, *p* < 0.001), subjective norm (*β* = 0.095, *p* < 0.05), and PBC (*β* = 0.579, *p* < 0.001). Attitude was not significantly associated with subjective norm (*r* = 0.093, *p* = 0.115) but positively and significantly associated with PBC (*r* = 0.588, *p* < 0.001). Subjective norm was positively but not significantly associated with PBC (*r* = 0.059, *p* = 0.333). The proposed model accounted for 67.9% of the variance in teachers’ intention to use CBP and for 39.7% of the variance in teachers’ CBP.

Concerning DI, the standardized regression paths from intention to behavior (*β* = 0.554, *p* < 0.001) was statistically significant while the one from PBC to behavior was not (*β* = 0.030, *p* = 0.412). The statistically significant predictors of intention regarding DI were attitude (*β* = 0.567, *p* < 0.001), subjective norm (*β* = 0.315, *p* < 0.001), and PBC (*β* = 0.327, *p* < 0.001). Attitude was positively but not significantly associated with subjective norm (*r* = 0.098, *p* = 0.253), and positively and significantly associated with PBC (*r* = 0.300, *p* < 0.001). Subjective norm was positively but not significantly associated with PBC (*r* = 0.068, *p* = 0.460). The model accounted for 68.7% of the variance in teachers’ intention to use DI and, respectively, for 32.5% of the variance in teachers’ DI practices.

Concerning FA, the standardized estimate from intention to behavior (*β* = 0.200, *p* < 0.01) and from PBC to behavior (*β* = 0.278, *p* < 0.001) were positive and statistically significant. The significant predictors of intention were attitude (*β* = 0.352, *p* < 0.001) and PBC (*β* = 0.568, *p* < 0.001). Subjective norm was positively but not significantly associated with attitude (*r* = 0.057, *p* = 0.362) and significantly and negatively with PBC (*r* = −0.129, *p* < 0.05). Attitude was significantly and positively associated with PBC (*r* = 0.451, *p* < 0.001). The model accounted for 62.4% of the variance in teachers’ intention to use FA and for 19.7% of the variance in teachers’ FA practices.

### Direct and indirect effects

Estimation of the indirect effect of a variable on another one through one mediator variable is useful because it can help to better understand the causal mechanisms. The direct and indirect effects of the predictors regarding CBP, DI, and FA are presented in Table 6.

**Table 6:** Direct and indirect effects of the TPB predictors on CBP/DI/FA practices.

		Attitude	Subjective norm	PBC	Intention
CBP	Direct effect (SE)	-	-	0.232 (.079)**	0.433 (0.077)**
	Indirect effect (SE)	0.139 (0.031)**	0.041 (0.022)	0.251 (0.041)**	-
	**Total effect (SE)**	0.139 (0.031)**	**0.041 (0.022)**	0.483 (0.041)**	0.433 (0.077)**
DI	Direct effect (SE)	-	-	0.030 (0.037)	0.554 (0.033)**
	Indirect effect (SE)	0.314 (0.033)**	0.174 (0.054)**	0.181 (0.027)**	-
	**Total effect (SE)**	0.314 (0.033)**	0.174 (0.054)**	0.211 (0.036)**	0.554 (0.033)**
FA	Direct effect (SE)	-	-	0.278 (0.065)**	0.200 (0.064)**
	Indirect effect (SE)	0.070 (0.025)**	0.011 (0.011)	0.114 (0.036)**	-
	**Total effect (SE)**	0.070 (0.025)**	**0.011 (0.011)**	0.392 (0.040)**	0.200 (0.064)**

**p < 0.01, *p < 0.05.

Table 6 shows that PBC had the highest total effect on CBP/DI/FA practices. Concerning CBP and FA practices, the total effects are made up of a statistically significant direct effect (0.232 and 0.278, respectively), and a statistically significant indirect effect through behavioral intention (0.251 and 0.114, respectively). Concerning DI, the direct effect of PBC is not statistically significant. Concerning CBP/DI/FA practices, attitude had a significant but low to moderate indirect effect (0.139, 0.314, and 0.070 respectively), while indirect effects of subjective norm via intention were non-significant, except for DI.

### Controlling for teachers’ experience, gender, teaching cycle, and socio-economic level of the school population

Four exogenous predictors have been added to the previous SEM models: teachers’ experience (in years), gender (0 = Female, 1 = Male), school level (1 = Cycle 1, 2 = Cycle 2, 3 = Cycle 3, 4 = Cycle 4, 5 = More than one cycle), and school proportion of socio-culturally and economically disadvantaged children (1 = Over 80%, 2 = Between 60 and 80%, 3 = Between 40 and 60%, 4 = Between 20 and 40%, 5 = 20% or less). Standardized estimates of these models are reported in of Supplementary Appendix 6.

Concerning CBP, results showed that the more experienced teachers significantly reported less frequent use of the behavior (*β* = −0.193, *p* < 0.01), less positive attitude (*β* = −0.175, *p* < 0.01), less influential subjective norm (*β* = −0.234, *p* < 0.01), and lower PBC (*β* = −0.082, *p* < 0.05). Men reported significant less positive attitude (*β* = −0.119, *p* < 0.01) than women. Compared to cycle 2 teachers, cycle 1 teachers reported more positive attitude toward CBP (*β* = 0.123, *p* < 0.01). Compared to schools with social and cultural mix, teachers working in schools with more than 80% of socio-culturally and economically disadvantaged children reported less positive attitude (*β* = −0.102, *p* < 0.05), less influential subjective norm (*β* = −0.153, *p* < 0.05) and lower PBC (*β* = −0.087, *p* < 0.05). Subjective norm was considered as more influential by teachers working in schools with social and cultural mix than those in schools with 20–40% of socio-culturally and economically disadvantaged children (*β* = −0.133, *p* < 0.05). With the addition of the four covariables, explained variance raised from 39.7 to 43.6% concerning CBP use, and slightly decrease from 67.9 to 67.3% concerning intention to implement CBP.

Concerning DI, results showed that men reported less intention to adopt the behavior (*β* = −0.127, *p* < 0.05), and less positive attitude (*β* = −0.108, *p* < 0.05) compared to women. The more experienced teachers reported less frequent use of DI practices (*β* = −0.104, *p* < 0.05) and less influential subjective norm (*β* = −0.226, *p* < 0.01). Compared to cycle 2 teachers, cycle 1 teachers reported less influential subjective norm (*β* = −0.313, *p* < 0.01). Compared to schools with social and cultural mix, teachers working in schools with more than 80% of socio-culturally and economically disadvantaged children reported less positive attitude (*β* = −0.114, *p* < 0.01) and lower PBC (*β* = −0.081, *p* < 0.05). Compared to schools with social and cultural mix, teachers working in schools with less than 20% of socio-culturally and economically disadvantaged children reported less influential subjective norm (*β* = −0.216, *p* < 0.05). With the addition of the four covariables, explained variance raised from 32.5 to 34.6% concerning DI behavior, and from 68.7 to 74.4% concerning intention to implement DI.

Concerning FA, results showed that the more experienced teachers reported again positive attitude (*β* = −0.222, *p* < 0.01), less influential subjective norm (*β* = −0.204, *p* < 0.01), and lower PBC (*β* = −0.107, *p* < 0.01). Compared to cycle 2 teachers, cycle 1 teachers reported a less frequent use FA (*β* = −0.155, *p* < 0.01) and lower PBC (*β* = −0.096, *p* < 0.05). Compared to schools with social and cultural mix, teachers working in schools with more than 80% of socio-culturally and economically disadvantaged children reported lower PBC (*β* = −0.112, *p* < 0.01). With the addition of these four covariables, explained variance raised from 19.7 to 23.3% concerning FA practices and from 62.4 to 65.3% concerning FA intention.

## Discussion

The present study aimed to examine the network of relationships among TPB variables and to pinpoint potential determinants influencing the adoption of CBP, DI, and FA practices in Luxembourgish elementary school. Three hypotheses were formulated.

Research *H*1: The full two-component version of the TPB provides a significantly better fit to the data, and accounts for a higher proportion of behavior variance than the original one-component version of the TPB (Ajzen, 1991).

As stated in the introduction, the TPB has evolved over the years. Studies that have used this theoretical framework have sometimes employed the full original one-component version (attitude, subjective norm, PBC) of the model, sometimes the full two-component version (instrumental attitude, affective attitude, injunctive norm, descriptive norm, controllability, self-efficacy) of the model, and often a hybrid version of the model. In the present study, we contrasted several CFA models to find the best TPB measurement model applied to CBP/DI/FA practices. Results first showed that fit indices for the full two-component models were good, but the distinction between instrumental attitude and affective attitude was not empirically based, as the two facets were highly correlated. As fit indices for the hybrid and full one-component models were all good, we finally selected the full one-component models for parsimony purpose. Research hypothesis 1 was then not confirmed.

Explained variance of the full one-component SEM models regarding intention and behavior were relatively high (between 60 and 70% for intention to use CBP/DI/FA practices and between 20 and 40% for behaviors). For comparison purposes, in Armitage and Conner (2001) TPB studies meta-analysis, an average of 39% of the variance in intention and an average of 27% of the variance in behavior were calculated. The proportions of variance explained in the present study are thus respectable, especially when the behaviors we are trying to explain (essentially from subjective psychological determinants) are working behaviors which are maybe more easily influenced by the surrounding context than personal behaviors (Yan and Cheng, 2015; Wilson et al., 2016).

Research *H*2: Teachers’ CBP, DI and FA practices are directly predicted by intention and perceived behavioral control regarding these practices.

In accordance with the TPB, the use of CBP/DI/FA practices was directly and positively predicted by intention. In other words, higher levels of intention were associated with more frequently reported implementation of CBP/DI/FA practices. Concerning PBC (measured here with a set of controllability and self-efficacy items), divergent results were observed depending on the practices under consideration. While the TPB assumes a positive significant direct association between PBC and behavior, the direct path from PBC toward DI practices was not statistically significant. In fact, the large effect of PBC regarding the use of DI practices was mainly indirect through intention. Concerning CBP, our results are in line with the TPB theory and the findings of Lenski et al. (2019) who considered only self-efficacy as a measure of PBC but reported a significant direct effect of intention and self-efficacy on competency-based instruction. Concerning DI, our results are in line with the first model tested by Hellmich et al. (2019), showing that DI practices were significantly and directly explained by intention, but not by collective self-efficacy beliefs. In the two models tested by Knauder and Koschmieder (2019), intention and self-efficacy were significantly associated with individualized support practices. Concerning FA, our results are in line with the TPB theory but differ from previous studies of Yan and Cheng (2015) and Karaman and Sahin (2017). In Yan and Cheng (2015) study, formative practices were significantly and directly predicted only by intention and not by controllability and self-efficacy. In Karaman and Sahin (2017) study, formative practices were directly and positively predicted by self-efficacy, but not significantly predicted by intention.

Research *H*3: Teachers’ intention to use CBP, DI and FA practices is directly predicted by attitude, subjective norm, and perceived behavioral control regarding these practices.

In line with the TPB theory, attitude, subjective norm, and PBC were significant predictors of the intention to use CBP and DI practices. Regarding FA, both attitude and PBC emerged as significant predictors of intention, whereas subjective norm did not show significance.

Concerning CBP, our results are in line with those of Lenski et al. (2019) who observed significant associations between attitude, subjective norm, PBC on one side, and intention on the other side. Concerning DI, our results are also in line with those of Hellmich et al. (2019) showing that attitude, subjective norm (i.e., school management expectations) and self-efficacy were significant predictors of intention. Our results are partly in line with those obtained by Knauder and Koschmieder (2019) who showed that the only significant predictor of intention was attitude. Concerning FA, our results differ from those of Yan and Cheng (2015) who observed that the significant predictors of intention were instrumental attitude, subjective norm, and self-efficacy, but not affective attitude and controllability. In Karaman and Sahin (2017) study, the standardized regression weights of the paths from instrumental attitude, self- efficacy, and controllability, to intention were statistically significant, but not those from affective attitude and subjective norm.

In summary, our findings and those from previous TPB studies conducted in the field of teaching practices are somewhat inconsistent. Results rarely fully support Ajzen’s TPB (whatever the version of the model) when teaching practices are examined. In some studies, the TPB model explained however a large amount of variance, both for intention and for behavior, suggesting that the theory is adequate. The originality of the present study was to apply the TPB to three pedagogical practices in a unique research design with similar measurement scales. Even under these conditions, there are contrasted explanatory configurations, suggesting that TPB results depend mainly on the targeted behavior.

### Implications for research

This quantitative study used the TPB to identify factors which may enable or hinder the use of recommended teaching practices. The SEM models predicted a considerable amount of the variance of self-reported practices, but a large part (60–80%) of this variance remains unexplained. Further quantitative research is needed to confirm the present findings and to explore other factors influencing the use of CBP, DI, and FA practices. In particular, it would be interesting to explore the potential impact of school-level policies which aim at encouraging and supporting teachers in the use of specific teaching practices.

Results of recent studies offered some avenues. For example, Bingham et al. (2021) conducted a qualitative case study and showed that teachers who faithfully implemented the competency-based approach made fundamental changes to their view of teaching practices. Rather than acting as conveyors and assessors of knowledge, they had to construct their classrooms in a way that engaged and supported students in owning their own learning. They also showed that teachers implementing competency-based approach encountered some considerable challenges around time investment (which was often insufficient to plan and to analyze students’ data), communication of new practices (in particular with parents), and alignment (with national curriculum and tests). The difficulty in alignment with the curriculum could indeed be one of the main reasons why teachers do not take up the competency-based approach. While teachers have at least a moral obligation to complete the teaching of the curriculum, the implementation of the competency-based approach (i.e., working from complex and significant problems in which students must find or build their own tools) seems more time-consuming and complicated than directly giving tools to students. Implementation time could therefore restrict the ability to complete the curriculum and induce teachers to work in a more transmissive manner, with the risk of making students passive receivers of information and not actors of their learning. Adopting the competency-based approach could also have a greater impact regarding preparation time. This is particularly the case for more experienced teachers compared with younger ones. Since it implies a profound change in their daily practices, this could explain why experience is negatively associated with attitude, subjective norm, or PBC in the present study. The more experienced is the teacher, the less they have been exposed to this practice as a student or teacher. We hypothesize that the implementation of a new way of working requires greater effort and a more intensive transformation for teachers with more experience.

Whitley et al. (2019) showed that teachers who had greater organizational school support (i.e., time to plan in collaborative ways, time to learn about DI, availability of technological resources) also had a higher DI self-efficacy and more positive DI beliefs, which were both directly associated with more frequent use of DI practices. This raises the importance of the environmental support needed for implementing all facets of DI, undoubtedly with more human resources (i.e., teaching assistants, team teaching), more advanced professional development, additional preparation time, more flexibility in scheduling and forming learning groups, and additional teaching time to intensively support struggling students.

Regarding FA, Schildkamp et al. (2020) identified a series of teacher prerequisites. Interestingly, they conducted a review distinguishing two approaches of formative assessment (data-based decision making, DBDM, and assessment for learning, AfL) which could nevertheless be complementary approaches. They identified three kinds of factors associated with use of DBDM and AfL: (1) knowledge and skills (adequate levels of data literacy, assessment literacy, pedagogical content knowledge, skills with regard to goal setting, providing feedback, facilitating classroom discussion, and ICT skills), (2) psychological factors (attitude toward formative assessment, ownership over the process and results of formative assessment, perceived control and autonomy), and (3) social factors (relationships between teachers, relationships between teachers and students, collaboration, students’ involvement).

In another systematic review on factors influencing teachers’ intentions and implementations regarding formative assessment, Yan et al. (2021) covered 52 studies identifying personal and contextual determinants of formative assessment. They concluded that the following factors were the most common predictors of teachers’ formative assessment practices: education and training, instrumental attitude, beliefs about teaching, school environment, internal school support, and working conditions. We think that further research should consider extended TPB models, including other measures of social influence, collective dynamics, and school environment that could better explain individual use of CBP/DI/FA practices in the classroom.

### Implications for teaching and professional development

The present study showed that teachers who use CBP, DI, and FA practices most frequently are those who have the higher motivational intention to adopt these practices in their classroom which, in turn, seems highly influenced by PBC and attitude. If we could prove that these associations are causal– which we cannot demonstrate with the present cross-sectional study –, efforts should be made in the direction of teachers’ professional development. The aim is indeed that teachers develop their knowledge and practical skills regarding CBP, DI, and FA, most preferably in real teaching situations, with the support of pedagogical experts or more experienced colleagues. We believe that this kind of support is of greater importance to avoid negative professional experiences, which could lead to a lower teacher self-efficacy or a less positive attitude, while trying alone to implement news ways of teaching. Results also suggested that experienced teachers had less positive attitude toward these practices. It would be interesting to know whether this result can be explained by younger teachers having been immersed in these practices during their schooling and training, or whether experienced teachers are less positive because they have greater hindsight on the real effectiveness of the practice or on the investment to make. It may also be interesting to analyze this result in the light of classical and more recent conceptual change theories (e.g., Posner et al., 1982; Vosniadou, 2007). When a reform is imposed, the conditions for accommodation are not systematically met because experienced teachers are possibly satisfied with the existing concepts that they have appropriated for many years and are therefore perhaps less open to conceptual change. If we attempt an analogy with Vosniadou’s vision of students’ difficulties in transferring school knowledge, official recommended practices (or even research-evidence-based practices) may be difficult to learn and to implement for teachers because it represents a different explanatory framework from the “naïve” theories teachers have implicitly constructed on the basis of their initial training, but above all, on the basis of their teaching experience.

As suggested by Lenski et al. (2019), the multilevel nature of educational effectiveness should be better considered. More precisely, they referred to the Dynamic model of educational effectiveness (Creemers and Kyriakides, 2008) which aims to explain different types of student outcomes (cognitive, affective, psychomotor, metacognitive), based on factors at the level of the student, teacher, school, and system. On this basis, promoting competency-based instruction could simultaneously entail the following specific actions: (1) a teacher training at the level of the classroom in order to learn about the competencies outlined in the educational standards, and how to create corresponding classroom activities, (2) a promotion and support of the competency-based approach by administrators at the school level, and (3) a nationwide support system for in-classroom implementation of educational standards and competency-based instruction.

In the case of teachers who appear to have little commitment to FA practices, it might be interesting to provide them with formative assessment tools that are psychometrically validated regarding the key curriculum knowledge and skills. In many cases, despite spending considerable time on this, teachers develop assessments that are not sufficiently valid and reliable in terms of measurement. We believe that providing validated formative assessment tools could encourage the use of formative assessment. These tools could also offer an analysis of the most common errors made by pupils, or even didactic avenues that could be implemented by teachers to overcome learning difficulties.

Finally, in TPB studies, attitude toward behavior is generally a strong predictor of the intention to adopt the behavior, and suggested ways to improve the adoption or the frequency of the behavior often consist of describing its positive consequences. In the field of teaching practices, we do not believe that working on attitude would be a solution to increase motivation and practices; most of the teachers are already aware of the benefit of such recommended practices. As suggested by Yan and Cheng (2015) and Schildkamp et al. (2020), the question is more to lead teachers to consider and apply CBP, DI, and FA practices as an integrated or interactive part of their regular instruction. This would be rather than an add-on activity that is retroactive and competes with other components for teaching time.

## Limitations and conclusion

The present study has several limitations. First, as data were exclusively collected through teachers’ self-reporting, findings of the present study might be vulnerable to response biases, such as social desirability or potential common source bias (Podsakoff et al., 2012). The survey was one of the rare opportunities given to Luxembourgish teachers in elementary school to anonymously give their opinion on the reforms undertaken. For this reason, we believe that the data are not significantly affected by social desirability bias. It would be useful for a future study to collect and examine both self-reported data and observational data regarding the use of CBP, DI, and FA practices. The convenient sample is a second limit of the study because we cannot exclude the possibility that the participants were the more motivated teachers, suggesting that the actual use of CBP, DI, and FA practices in Luxembourgish elementary classrooms is maybe overestimated. It is therefore not possible to generalize the findings from this sample to the population of elementary school teachers in Luxembourg. The results cannot be generalized beyond this specific sample. Third, the specific Luxembourgish context where CBP, DI, and FA practices are mandatory by law could have influenced the findings of the study. Replication studies should be conducted in educational systems where teachers are free to decide how they want to teach. The imposition of such reforms may limit teachers’ ability to exercise professional judgment and adapt teaching strategies based on their expertise and the unique needs of their students. This could potentially resulting in compliance rather than meaningful, sustainable change in teaching practices. Fourth, the correlational nature of the data prevents us from establishing causal relationships between the TPB variables. Longitudinal research designs are absolutely essential. Lastly, further studies could improve some of the measurement scales used in the present study and test for measurement invariance with larger samples.

In summary, the present study showed that the frequency of CBP, DI, and FA practices is positively associated with intention to implement these practices, and rather indirectly by PBC. The motivational component (i.e., intention), which is somewhat well explained by attitude and PBC, appears to be the cornerstone of the explanatory model, but a significant proportion of the variance observed in practices remains unexplained, suggesting that other important factors remain hidden. Further research should also examine the extent to which CBP, DI, and FA practices are used uniformly by elementary teachers, or whether they depend, for example, on school subjects.

## Data availability statement

The raw data supporting the conclusions of this article will be made available by the authors, without undue reservation.

## Ethics statement

This study involving humans was approved by the Ethics Review Panel (University of Luxembourg, ERP_21_034_CEEF). The study was conducted in accordance with the local legislation and institutional requirements. The participants provided their written informed consent to participate in this study.

## Author contributions

CD: Writing – original draft, Writing – review & editing, Conceptualization, Data curation, Formal analysis. DP: Writing – review & editing. MT-V: Conceptualization, Data curation, Writing – review & editing.

## Footnotes

^1^For a description of the country and the elementary education system, as well as official recommendations concerning teaching practices, please consult Appendix 1 in Supplementary material.

^2^As shown by Raykov and Marcoulides (2011), ordinal measures can nevertheless be treated as continuous measures if they have at least five response options, and if there is no piling of respondents at the extreme response options. Moreover, some specific estimator (i.e., WSLMV) can be used in SEM softwares to deal with ordinal data.
