# Supplementary material for: Why teachers do (or do not) implement recommended teaching practices? An application of the theory of planned behavior
Source: Front Psychol. 2024 Mar 13;15:1269954. doi: 10.3389/fpsyg.2024.1269954 (PMC10966465; doi:10.3389/fpsyg.2024.1269954)
Supplement: Supplementary file 1 [file Data_Sheet_1.docx]

***Online Supplements for:***

**Why teachers do (or do not) implement recommended teaching practices?**

**An application of the Theory of Planned Behavior in the Luxembourgish school system**

Appendix 1: Luxembourgish school context and official recommended teaching practices

The Grand Duchy of Luxembourg is a small Western Europe country (around 645000 residents, 47% of foreigners) bordered by Germany, France, and Belgium. Luxembourg is officially a trilingual country (Luxembourgish is the national language, French is the legislative language and French, German and Luxembourgish are the three administrative and judicial languages) but languages spoken by foreigners (such as Portuguese, Italian, English, and Slavic languages) are widely used. The country has 157 elementary public schools (around 50000 pupils) which are under the management and pedagogical supervision of 15 regional directors. In elementary schools, there is no principals but a school committee including 3 to 9 school members elected by the school staff, one of whom is elected as school president. There are 4 two-year learning cycles (C1, C2, C3, C4) in the Luxembourgish elementary school system, with the possibility to have an additional third year at the end of each cycle in case of severe learning difficulties.

The general objectives of elementary schooling are defined in Article 6 of the Law: “*Elementary schooling aims to develop progressively in pupils: (1) language, mathematical and scientific knowledge, and competences* […] *in order to make them fit for further education and lifelong learning*”. Competency is defined as “*the ability to perform a task based on a set of acquired knowledge, skills and attitudes*” and competency thresholds (to be achieved at each cycle) are formulated in the curriculum.

The obligation to use differentiated instruction is defined in Article 9 and Article 22 of the 2009 Law. Art. 9. “*The teacher's mission is to help his/her pupils to achieve the objectives defined in the curriculum by means of pedagogical differentiation measures*”. Art. 22. “*In order to enable pupils to achieve the objectives set by the curriculum within the time allowed, the teaching teams rely on the following devices and measures of pedagogical differentiation: (1)Differentiation of learning paths within the class to help pupils who are experiencing difficulties and to stimulate pupils who show high aptitudes; (2)Decompartmentalization measures consisting in allowing students from different classes to be grouped together temporarily according to their needs, interests, or level of competence, (3)The possibility for a pupil to follow lessons in another cycle, (4) Support measures decided at the end of the cycle to be implemented in the next cycle according to the needs of the pupil.”*

Formative assessment is defined in Article 24 of the Law: “*Evaluation is at the service of learning. Its objectives are (1) observation of the student's work and adaptation of teaching to his/her needs, (2) to inform the pupil, his/her parents and the staff involved on a regular basis about progress made, and (3) to make informed decisions in relation to the pupil's progress during and at the end of the cycle*”. The Règlement grand-ducal du 6 juillet 2009 specified that: “*During a learning cycle, assessment is formative. Formative assessment is based on the following principles:* (*1) It gives each student the opportunity to show what they know and can do, (2) It focuses on the mobilization of skills in concrete situations rather than on the assimilation and reproduction of isolated knowledge, (3) It takes into account the different ways in which students learn and the differences between students in terms of their cognitive, language, motor, emotional and social development, and (4) It enables students to be accountable for their progress: it encourages them to question their progress, to explain and document their learning process and thinking strategies.*

Appendix 2: Observed proportions, descriptive statistics, and standardized CFA estimates for items related to competency-based practices (N=952)

|  | | 999 | -999 | n | Mean (sd) | 1 | 2 | 3 | 4 | 5 | 6 | 7 | 777 | 888 | λ (SE) |
| --- | --- | --- | --- | --- | --- | --- | --- | --- | --- | --- | --- | --- | --- | --- | --- |
| ATTITUDE | |  |  |  |  |  |  |  |  |  |  |  |  |  |  |
| *Instrumental facet* | |  |  |  |  |  |  |  |  |  |  |  |  |  |  |
| CA1 | The competency-based approach is an effective way to improve student engagement. | 773 | - | 179 | - | 5 | 7.3 | 14 | 33.5 | 19 | 4.5 | - | 11.2 | 5.6 | .85 (.03)** |
| CA2 | Teaching using a competency-based approach is useful. | 0 | - | 952 | - | 1.1 | 5 | 8.9 | 35.8 | 31.3 | 9.5 | - | 5.6 | 2.8 | .87 (.02)** |
| CA3 | The competency-based approach is an effective way to improve school learning. | 0 | - | 952 | - | 2.8 | 5.7 | 11.3 | 36.9 | 28.4 | 5.9 | - | 6 | 3 | .88 (.01)** |
| *Affective facet* | |  |  |  |  |  |  |  |  |  |  |  |  |  |  |
| CA4 | The competency-based approach is a bad idea. ^R^ | 0 | - | 952 | - | 17.5 | 33.9 | 27.3 | 6.1 | 3.9 | 2.5 | - | 6.6 | 2.1 | -.84 (.01)** |
| CA5 | Teaching according to the competency-based approach is professionally satisfying. | 773 | - | 179 | - | 3.3 | 6.6 | 12 | 35.8 | 22.5 | 4.2 | - | 10.6 | 5 | .83 (.01)** |
| CA6 | The competency-based approach is demotivating. ^R^ | 0 | - | 952 | - | 13.4 | 34.1 | 31.3 | 3.9 | 5.6 | 1.1 | - | 6.1 | 4.5 | -.87 (.03)** |
| SUBJECTIVE NORM | |  |  |  |  |  |  |  |  |  |  |  |  |  |  |
| *Injunctive facet* | |  |  |  |  |  |  |  |  |  |  |  |  |  |  |
| CN1 | I am expected to implement the competency-based approach to a greater extent. | 0 | - | 952 | - | 5.8 | 13.6 | 16 | 23.6 | 20.4 | 9.9 | - | 8.3 | 2.5 | .60 (.07)** |
| CN2 | The regional director encourages me to further develop the competency-based approach. | 773 | - | 179 | - | 3.4 | 12.8 | 13.4 | 24 | 17.3 | 5 | - | 20.1 | 3.9 | .68 (.07)** |
| CN3 | I feel a certain amount of social pressure (from regional direction, colleagues and/or parents) to further develop the competency-based approach. | 0 | - | 952 | - | 8.2 | 17.9 | 24.1 | 17 | 14.7 | 6.5 | - | 8.7 | 2.9 | - |
| *Descriptive facet* | |  |  |  |  |  |  |  |  |  |  |  |  |  |  |
| CN4 | In my cycle, the competency-based approach is at the heart of teaching practices. | 0 | - | 952 | - | 2.8 | 5.1 | 12 | 31.4 | 28.9 | 10.3 | - | 6.8 | 2.6 | - |
| CN5 | My practices with regard to the competency-based approach are influenced by the practices implemented in the other classes of the cycle or the school. | 773 | - | 179 | - | 4.5 | 11.2 | 17.3 | 27.4 | 26.3 | 3.4 | - | 6.7 | 3.4 | .70 (.06)** |
| CN6 | The cycle meetings with my colleagues are pushing me to develop my own practices in terms of the competency-based approach. | 773 | - | 179 | - | 6.7 | 15.6 | 23.5 | 22.9 | 16.8 | 3.4 | - | 10.1 | 1.1 | .67 (.05)** |
| PERCEIVED BEHAVIORAL CONTROL | |  |  |  |  |  |  |  |  |  |  |  |  |  |  |
| *Controllability* | |  |  |  |  |  |  |  |  |  |  |  |  |  |  |
| CC1 | It is up to me to develop my practices further in line with the competency-based approach. | 0 | - | 952 | - | 2.4 | 11.9 | 18.8 | 27.6 | 18.6 | 4.7 | - | 10.4 | 5.6 | .26 (.03)** |
| CC2 | I decide for myself whether or not to develop my practices further in line with the competency-based approach. | 773 | - | 179 | - | 0.6 | 6.7 | 15.1 | 32.4 | 25.1 | 5.6 | - | 11.7 | 2.8 | - |
| CC3 | The current conditions in which I work do not allow me to implement the competency-based approach. ^R^ | 773 | - | 179 | - | 6.1 | 26.3 | 24.6 | 21.8 | 7.3 | 2.8 | - | 6.7 | 4.5 | -.51 (.06)** |
| CC4 | I don't have enough resources to implement the competency-based approach as I conceive it. ^R^ | 0 | - | 952 | - | 4.2 | 14.5 | 23.7 | 21.8 | 17.2 | 7.5 | - | 8.3 | 2.7 | -.30 (.03)** |
| *Self-efficacy* | |  |  |  |  |  |  |  |  |  |  |  |  |  |  |
| CS1 | Implement the competency-based approach | - | 60 | 892 | 3.9 (1.1) | - | - | - | - | - | - | - | - | - | .88 (.01)** |
| CS2 | Encourage students to transfer their learning to other situations. | 773 | 6 | 173 | 4.0 (0.9) | - | - | - | - | - | - | - | - | - | .76 (.04)** |
| CS3 | Provide pupils with complex and/or problematic learning situations that justify the acquisition of new knowledge. | 773 | 9 | 170 | 3.9 (1.0) | - | - | - | - | - | - | - | - | - | .83 (.03)** |
| CS4 | Develop students' ability to mobilise appropriate resources in response to a problem. | - | 61 | 891 | 4.0 (0.9) | - | - | - | - | - | - | - | - | - | .71 (.02)** |
| INTENTION | |  |  |  |  |  |  |  |  |  |  |  |  |  |  |
| CI1 | To implement competency-based approach. | - | 49 | 903 | 4.0 (1.3) | - | - | - | - | - | - | - | - | - | .89 (.01)** |
| CI2 | To develop pupils' ability to transfer their knowledge, skills and attitudes to other situations. | - | 31 | 921 | 4.5 (1.1) | - | - | - | - | - | - | - | - | - | .65 (.02)** |
| CI3 | To place pupils in complex and/or problem learning situations that justify the acquisition of new knowledge. | - | 41 | 911 | 4.3 (1.2) | - | - | - | - | - | - | - | - | - | .67 (.02)** |
| BEHAVIOR | |  |  |  |  |  |  |  |  |  |  |  |  |  |  |
| CB1 | I place students in complex and/or problem learning situations that justify the acquisition of new knowledge. | 0 | - | 952 | - | 0.3 | 3.7 | 18.5 | 27.4 | 20.6 | 14.7 | 9 | 4.2 | 1.6 | .80 (.01)** |
| CB2 | I implement the competency-based approach. | 0 | - | 952 | - | 0.6 | 2.2 | 10.6 | 25.7 | 20.1 | 18.7 | 17.4 | 2.8 | 1.8 | .84 (.01)** |
| CB3 | I organize lessons/activities where students practice transferring their knowledge, skills and attitudes to other situations. | 0 | - | 952 | - | 0.3 | 3.3 | 17.2 | 27.5 | 22.9 | 14.1 | 10.8 | 3.2 | 0.7 | .82 (.01)** |
| CB4 | I place the pupils in problem-solving situations. | 773 | - | 179 | - | 0.6 | 3.9 | 20.7 | 29.6 | 22.9 | 15.1 | 4.5 | 2.8 | 0.0 | .80 (.03)** |
| CB5 | I provide a problem-based teaching/learning approach. | 773 | - | 179 | - | 1.1 | 5.6 | 24.6 | 30.2 | 16.2 | 12.3 | 3.4 | 4.5 | 2.2 | .80 (.04)** |

Note. 999=number of missing answers by design. -999=number of true missing. 888= number of “*I don’t know*” answers. 777=number of ”*Not applicable*” answers. For the attitude and subjective norm scales: 1=not at all agree, 2=disagree, 3=somewhat disagree, 4=somewhat agree, 5=agree, 6=strongly agree. For the controllability items in Perceived Behavioral Control: 1=not at all agree, 2=disagree, 3=somewhat disagree, 4=somewhat agree, 5=agree, 6=strongly agree. For the self-efficacy items in Perceived Behavioral Control: 0=not at all competent, 6=extremely competent. For the intention scale: 0=very little determined, 6=very determined. For the behavior scale: 1=never, 2=rarely, 3=sometimes, 4=regularly, 5=frequently, 6=very frequently, 7=systematically. ^R^ =Reverse coded item. λ: Standardized factor loading. * p ≤ .05. ** p ≤ .01.

Appendix 3: Observed proportions, descriptive statistics, and standardized CFA estimates for items related to differentiated instruction practices (N=952)

|  | | 999 | -999 | n | Mean (sd) | 1 | 2 | 3 | 4 | 5 | 6 | 7 | 777 | 888 | λ (SE) |
| --- | --- | --- | --- | --- | --- | --- | --- | --- | --- | --- | --- | --- | --- | --- | --- |
| ATTITUDE | |  |  |  |  |  |  |  |  |  |  |  |  |  |  |
| *Instrumental facet* | |  |  |  |  |  |  |  |  |  |  |  |  |  |  |
| DA1 | Differentiated instruction is an effective way to improve student engagement. | 810 | - | 142 | - | 1.4 | 2.8 | 9.2 | 24.6 | 35.9 | 22.5 | - | 0.7 | 2.8 | .76 (.05)** |
| DA2 | Implementing differentiated instruction practices is useful. | 810 | - | 142 | - | 0.0 | 0.0 | 2.1 | 16.2 | 38.7 | 43 | - | 0.0 | 0.0 | .87 (.05)** |
| DA3 | Differentiated instruction practices are effective in improving school learning. | 0 | - | 952 | - | 2 | 4.7 | 7.5 | 29 | 32.9 | 20.8 | - | 1.9 | 1.3 | .73 (.02)** |
| *Affective facet* | |  |  |  |  |  |  |  |  |  |  |  |  |  |  |
| DA4 | Differentiated instruction is generally a bad idea. ^R^ | 0 | - | 952 | - | 45.5 | 32.8 | 14.3 | 2.9 | 1.2 | 0.9 | - | 1.5 | 0.9 | -.82 (.02)** |
| DA5 | Implementing differentiated instruction practices is professionally satisfying. | 0 | - | 952 | - | 3.4 | 5.7 | 14.3 | 29.5 | 26.5 | 8.5 | - | 5.7 | 6.5 | .61 (.02)** |
| DA6 | Implementing differentiated instruction practices is demotivating. ^R^ | 810 | - | 142 | - | 21.8 | 33.1 | 29.6 | 4.2 | 6.3 | 0.7 | - | 2.8 | 1.4 | -.85 (.025)** |
| SUBJECTIVE NORM | |  |  |  |  |  |  |  |  |  |  |  |  |  |  |
| *Injunctive facet* | |  |  |  |  |  |  |  |  |  |  |  |  |  |  |
| DN1 | I am expected to implement differentiated instruction practices to a greater extent. | 0 | - | 952 | - | 3.6 | 10.5 | 15.2 | 23.3 | 23.1 | 16.3 | - | 6.6 | 1.4 | .24 (.08)** |
| DN2 | The regional director encourages me to further develop my differentiated instruction practices. | 810 | - | 142 | - | 6.3 | 11.3 | 19.7 | 23.9 | 22.5 | 4.9 | - | 10.6 | 0.7 | .41 (.09)** |
| DN3 | I feel a certain amount of social pressure (from regional direction, colleagues and/or parents) to further develop my differentiation instruction practices. | 0 | - | 952 | - | 6.6 | 16.8 | 21.6 | 19.2 | 17.8 | 13 | - | 4.1 | 0.8 | - |
| *Descriptive facet* | |  |  |  |  |  |  |  |  |  |  |  |  |  |  |
| DN4 | In my cycle, differentiated instruction is at the heart of teaching practices. | 0 | - | 952 | - | 0.9 | 3.5 | 11 | 31.3 | 31.7 | 18.2 | - | 2.3 | 1.1 | - |
| DN5 | My differentiated instruction practices are influenced by the practices implemented in the other classes of the cycle or the school. | 810 | - | 142 | - | 2.8 | 12 | 16.2 | 31 | 28.9 | 4.9 | - | 2.8 | 1.4 | .50 (.09)** |
| DN6 | The cycle meetings with my colleagues are pushing me to develop my own differentiated instruction practices. | 810 | - | 142 | - | 4.2 | 9.2 | 22.5 | 28.2 | 26.8 | 4.2 | - | 3.5 | 1.4 | .93 (.14)** |
| PERCEIVED BEHAVIORAL CONTROL | |  |  |  |  |  |  |  |  |  |  |  |  |  |  |
| *Controllability* | |  |  |  |  |  |  |  |  |  |  |  |  |  |  |
| DC1 | It is up to me to develop my practices further in line with differentiation instruction. | 0 | - | 952 | - | 3.2 | 12.3 | 19.4 | 25.9 | 24.9 | 6.6 | - | 5 | 2.6 | .27 (.04)** |
| DC2 | I decide for myself whether or not to develop my practices further in line with differentiation instruction. | 810 | - | 142 | - | 2.1 | 13.4 | 24.6 | 28.2 | 23.2 | 5.6 | - | 2.1 | 0.7 | - |
| DC3 | The current conditions in which I work do not allow me to implement differentiation instruction. ^R^ | 810 | - | 142 | - | 2.8 | 21.8 | 28.2 | 28.9 | 12.7 | 3.5 | - | 1.4 | 0.7 | -.37 (.07)** |
| DC4 | I don't have enough resources to implement differentiation instruction as I conceive it. ^R^ | 0 | - | 952 | - | 5 | 13.4 | 18.5 | 23.4 | 20.1 | 14.6 | - | 4.1 | 0.8 | -.28 (.04)** |
| *Self-efficacy* | |  |  |  |  |  |  |  |  |  |  |  |  |  |  |
| DS1 | Provide quality advice and education to all students, regardless of their level of ability. | - | 36 | 916 | 4.3 (0.9) | - | - | - | - | - | - | - | - | - | .70 (.02)** |
| DS2 | Explain the material in such a way that the large majority of students understand the basic principles. | 660 | 11 | 281 | 4.7 (0.9) | - | - | - | - | - | - | - | - | - | .72 (.03)** |
| DS3 | Organize schoolwork to adapt teaching and tasks to individual needs. | - | 43 | 909 | 4.1 (1.0) | - | - | - | - | - | - | - | - | - | .90 (.01)** |
| DS4 | Challenge all students to learn, even in a very heterogeneous class. | - | 43 | 909 | 3.9 (1.1) | - | - | - | - | - | - | - | - | - | .78 (.02)** |
| DS5 | Organize class work so that weak and strong students have tasks appropriate to their abilities. | 660 | 13 | 279 | 4.1 (1.0) | - | - | - | - | - | - | - | - | - | .96 (.03)** |
| INTENTION | |  |  |  |  |  |  |  |  |  |  |  |  |  |  |
| DI1 | To implement differentiated instruction practices. | - | 30 | 922 | 4.6 (1.1) | - | - | - | - | - | - | - | - | - | .89 (.01)** |
| DI2 | To implement differentiated instruction practices adapted to the needs of the pupils, according to the difficulties highlighted by the assessments. | - | 43 | 909 | 4.5 (1.2) | - | - | - | - | - | - | - | - | - | .85 (.01)** |
| DI3 | To adapt my teaching practices to the identified needs of the students. | - | 30 | 922 | 4.8 (1.0) | - | - | - | - | - | - | - | - | - | .79 (.01)** |
| BEHAVIOR | |  |  |  |  |  |  |  |  |  |  |  |  |  |  |
| DB1 | I use alternative materials to match students’ abilities (e.g. books adapted to the needs of lower and higher skill levels). | 0 | - | 952 | - | 1.1 | 3.2 | 11.6 | 19.7 | 17.2 | 19 | 25.2 | 2.7 | 0.3 | .82 (.01)** |
| DB2 | I plan different assignments to match students’ abilities. | 0 | - | 952 | - | 0.3 | 2 | 11.2 | 22.8 | 17.8 | 19 | 25.2 | 1.6 | 0.1 | .85 (.01)** |
| DB3 | I vary the complexity of assignments to match students’ abilities. (e.g. make judgments about a text or summarize, recognize the main theme). | 0 | - | 952 | - | 0.6 | 2.1 | 8.9 | 22.4 | 19.9 | 18.8 | 20.6 | 6 | 0.7 | .81 (.01)** |
| DB4 | I adapt evaluations to match students’ abilities (by proposing different questions or by modulating my level of requirement). | 0 | - | 952 | - | 1.9 | 4.9 | 16.4 | 19.6 | 17 | 15.2 | 19.6 | 5 | 0.2 | .79 (.01)** |
| DB5 | I adjust the amount of work required in accordance with students' capabilities. | 0 | - | 952 | - | 0.2 | 0.6 | 6.4 | 20.5 | 17.4 | 20.7 | 32.9 | 1.2 | 0.1 | .78 (.01)** |
| DB6 | I modify goals and expectations for students with difficulties. | 0 | - | 952 | - | 0.4 | 1.5 | 8 | 23.6 | 19.7 | 17 | 27.9 | 1.5 | 0.3 | .84 (.01)** |
| DB7 | I provide weaker students with additional aids and tools (e.g. visual aids containing essential information). | 0 | - | 952 | - | 0.2 | 0.7 | 7.1 | 21.2 | 15.4 | 22 | 31.3 | 1.9 | 0.1 | .83 (.01)** |
| DB8 | I evaluate the effectiveness of my teaching practices (for example, by monitoring results and progress). | 0 | - | 952 | - | 10.1 | 14 | 17.5 | 17.2 | 10.3 | 8.3 | 8.2 | 10.2 | 4.2 | .51 (.02)** |
| DB9 | I use the data collected about students' skills to decide what adjustments to make in my teaching. | 0 | - | 952 | - | 0.9 | 2.2 | 11.7 | 23 | 21.3 | 17.3 | 17.2 | 4.5 | 1.8 | .78 (.01)** |
| DB10 | I assess the progress of weak students. | 0 | - | 952 | - | 0.4 | 1.2 | 4.5 | 27.2 | 18.2 | 19.2 | 25.3 | 3.4 | 0.6 | .77 (.01)** |
| DB11 | I analyze data about students' academic progress. | 0 | - | 952 | - | 1.1 | 2.5 | 8.5 | 27.6 | 20.3 | 16.3 | 13.1 | 6.3 | 4.3 | .77 (.01)** |

Note. 999=number of missing answers by design. -999=number of true missing. 888= number of “*I don’t know*” answers. 777=number of ”*Not applicable*” answers. For the attitude and subjective norm scales: 1=not at all agree, 2=disagree, 3=somewhat disagree, 4=somewhat agree, 5=agree, 6=strongly agree. For the controllability items in Perceived Behavioral Control: 1=not at all agree, 2=disagree, 3=somewhat disagree, 4=somewhat agree, 5=agree, 6=strongly agree. For the self-efficacy items in Perceived Behavioral Control: 0=not at all competent, 6=extremely competent. For the intention scale: 0=very little determined, 6=very determined. For the behavior scale: 1=never, 2=rarely, 3=sometimes, 4=regularly, 5=frequently, 6=very frequently, 7=systematically. ^R^ =Reverse coded item. λ: Standardized factor loading. * p ≤ .05. ** p ≤ .01.

Appendix 4: Observed proportions, descriptive statistics, and standardized CFA estimates for items related to formative assessment practices (N=952)

|  | | 999 | -999 | n | Mean (sd) | 1 | 2 | 3 | 4 | 5 | 6 | 7 | 777 | 888 | λ (SE) |
| --- | --- | --- | --- | --- | --- | --- | --- | --- | --- | --- | --- | --- | --- | --- | --- |
| ATTITUDE | |  |  |  |  |  |  |  |  |  |  |  |  |  |  |
| *Instrumental facet* | |  |  |  |  |  |  |  |  |  |  |  |  |  |  |
| FA1 | Formative assessment is an effective way to improve student engagement. | 802 | - | 150 | - | 3.3 | 8.7 | 8.7 | 34 | 21.3 | 6 | - | 11.3 | 6.7 | .63 (.04)** |
| FA2 | Practicing formative assessment is useful. | 802 | - | 150 | - | 0.0 | 1.3 | 2.7 | 28 | 40.7 | 12.7 | - | 10 | 4.7 | .71 (.03)** |
| FA3 | Formative assessment is an effective way to improve learning in schools. | 0 | - | 952 | - | 1.6 | 3.9 | 8.2 | 33.7 | 31.6 | 10.6 | - | 7.7 | 2.7 | .89 (.01)** |
| *Affective facet* | |  |  |  |  |  |  |  |  |  |  |  |  |  |  |
| FA4 | Formative assessment is generally a bad idea. ^R^ | 0 | - | 952 | - | 22.6 | 32 | 26.5 | 4.9 | 2.8 | 1.4 | - | 7.5 | 2.3 | -.81 (.01)** |
| FA5 | Practicing formative assessment is professionally satisfying. | 0 | - | 952 | - | 2 | 4.1 | 11.2 | 33.7 | 25 | 6.3 | - | 12.2 | 5.5 | .80 (.01)** |
| FA6 | Practicing formative assessment is demotivating. ^R^ | 802 | - | 150 | - | 14 | 27.3 | 33.3 | 6 | 2.7 | 0.7 | - | 11.3 | 4.7 | -.85 (.03)** |
| SUBJECTIVE NORM | |  |  |  |  |  |  |  |  |  |  |  |  |  |  |
| *Injunctive facet* | |  |  |  |  |  |  |  |  |  |  |  |  |  |  |
| FN1 | I am expected to implement formative assessment to a greater extent. | 0 | - | 952 | - | 5.9 | 14.5 | 21.4 | 19.1 | 16 | 6.2 | - | 12.8 | 4.1 | .58 (.05)** |
| FN2 | The regional director encourages me to further develop my formative assessment practices. | 802 | - | 150 | - | 13.3 | 13.3 | 18 | 18.7 | 12 | 1.3 | - | 18 | 5.3 | .60 (.07)** |
| FN3 | I feel a certain amount of social pressure (from regional direction, colleagues and/or parents) to further develop my formative assessment practices. | 0 | - | 952 | - | 8.1 | 17.6 | 28.7 | 15.2 | 12 | 4.8 | - | 10.4 | 3.2 | - |
| *Descriptive facet* | |  |  |  |  |  |  |  |  |  |  |  |  |  |  |
| FN4 | In my cycle, formative assessment is at the heart of teaching practices. | 0 | - | 952 | - | 2.4 | 5.6 | 12.4 | 34.8 | 25.7 | 7.2 | - | 8.6 | 3.3 | - |
| FN5 | My formative assessment practices are influenced by the practices implemented in other classes in the cycle or school. | 802 | - | 150 | - | 8 | 12 | 16.7 | 26 | 19.3 | 3.3 | - | 9.3 | 5.3 | .61 (.05)** |
| FN6 | The cycle meetings with my colleagues are pushing me to develop my formative assessment practices. | 802 | - | 150 | - | 10 | 17.3 | 22 | 25.3 | 11.3 | 2 | - | 8 | 4 | .79 (.05)** |
| PERCEIVED BEHAVIORAL CONTROL | |  |  |  |  |  |  |  |  |  |  |  |  |  |  |
| *Controllability* | |  |  |  |  |  |  |  |  |  |  |  |  |  |  |
| FC1 | It is up to me to develop my practices further in line with formative assessment. | 0 | - | 952 | - | 2.3 | 8.8 | 17.8 | 31.2 | 23.2 | 4.7 | - | 8 | 4 | .28 (.03)** |
| FC2 | I decide for myself whether or not to develop my practices further in line with formative assessment. | 802 | - | 150 | - | 0.7 | 8 | 13.3 | 36.7 | 23.3 | 4.7 | - | 9.3 | 4 | - |
| FC3 | The current conditions in which I work do not allow me to implement formative assessment. ^R^ | 802 | - | 150 | - | 5.3 | 16.7 | 24.7 | 20.7 | 14 | 7.3 | - | 8.7 | 2.7 | -.52 (.07)** |
| FC4 | I don't have enough resources to implement formative assessment as I conceive it. ^R^ | 0 | - | 952 | - | 4.6 | 15.9 | 26.8 | 23 | 12.3 | 5.3 | - | 8.6 | 3.6 | -.41 (.03)** |
| *Self-efficacy* | |  |  |  |  |  |  |  |  |  |  |  |  |  |  |
| FS1 | Implement formative assessment. | - | 85 | 867 | 4.0 (1.1) | - | - | - | - | - | - | - | - | - | .86 (.02)** |
| FS2 | Propose remedial activities following an assessment. | - | 81 | 871 | 4.0 (1.0) | - | - | - | - | - | - | - | - | - | .71 (.02)** |
| FS3 | Analyze and use assessment data for formative purposes. | 802 | 18 | 132 | 3.9 (1.1) | - | - | - | - | - | - | - | - | - | .84 (.04)** |
| FS4 | Integrate formative assessment into teaching/learning situations (interactive regulation). | 802 | 21 | 129 | 3.9 (1.0) | - | - | - | - | - | - | - | - | - | .79 (.04)** |
| INTENTION | |  |  |  |  |  |  |  |  |  |  |  |  |  |  |
| FI1 | To practice formative assessment. | - | 76 | 876 | 4.1 (1.3) | - | - | - | - | - | - | - | - | - | .90 (.01)** |
| FI2 | To provide assessments that enable students to identify their strengths and weaknesses accurately. | - | 70 | 882 | 4.2 (1.3) | - | - | - | - | - | - | - | - | - | .65 (.02)** |
| FI3 | To offer assessments on which remediation or refresher activities are based. | - | 134 | 818 | 3.9 (1.3) | - | - | - | - | - | - | - | - | - | .67 (.02)** |
| BEHAVIOR | |  |  |  |  |  |  |  |  |  |  |  |  |  |  |
| FB1 | My assessments are truly formative for the students. | 0 | - | 952 | - | 3.2 | 8.4 | 13.6 | 20.6 | 16.2 | 14.4 | 15.7 | 6.9 | 1.2 | .49 (.02)** |
| FB2 | I analyze the most common mistakes made during assessments in order to identify precisely what needs to be worked on as a priority with all or some of the students. | 0 | - | 952 | - | 0.3 | 1.7 | 6.2 | 23.8 | 21 | 17.1 | 23.3 | 6.2 | 0.3 | .70 (.02)** |
| FB3 | I observe the students while they are doing a particular task in class and give them direct feedback on their work. | 0 | - | 952 | - | 0.2 | 0.6 | 4.7 | 17.9 | 17.8 | 22.6 | 35.2 | 0.8 | 0.2 | .61 (.02)** |
| FB4 | After each assessment, I provide feedback to the students on which aspects they have mastered well and on which aspects they need to make further efforts. | 0 | - | 952 | - | 1.1 | 2.9 | 10.7 | 21 | 17.2 | 16.6 | 25 | 5 | 0.4 | .74 (.01)** |
| FB5 | After each assessment, I give additional explanations to students who are struggling. | 0 | - | 952 | - | 0.8 | 1.8 | 11.1 | 22.9 | 19.6 | 19.2 | 18.6 | 5.4 | 0.5 | .78 (.01)** |
| FB6 | After each assessment, I design remedial activities (set up a corrective procedure) for students in difficulty. | 0 | - | 952 | - | 1.1 | 5.8 | 18.7 | 26.1 | 16.7 | 13.1 | 10 | 6.9 | 1.2 | .71 (.02)** |

Note. 999=number of missing answers by design. -999=number of true missing. 888= number of “*I don’t know*” answers. 777=number of ”*Not applicable*” answers. For the attitude and subjective norm scales: 1=not at all agree, 2=disagree, 3=somewhat disagree, 4=somewhat agree, 5=agree, 6=strongly agree. For the controllability items in Perceived Behavioral Control: 1=not at all agree, 2=disagree, 3=somewhat disagree, 4=somewhat agree, 5=agree, 6=strongly agree. For the self-efficacy items in Perceived Behavioral Control: 0=not at all competent, 6=extremely competent. For the intention scale: 0=very little determined, 6=very determined. For the behavior scale: 1=never, 2=rarely, 3=sometimes, 4=regularly, 5=frequently, 6=very frequently, 7=systematically. ^R^ =Reverse coded item. λ: Standardized factor loading. * p ≤ .05. ** p ≤ .01.

Appendix 5: Extract of the planned missing data design concerning CBP, DI and FA items

|  |  | Version 1 | Version 2 | Version 3 | Version 4 | Version 5 | Version 6 |
| --- | --- | --- | --- | --- | --- | --- | --- |
| CBP items | CA1 | X |  |  |  |  |  |
|  | CA2 | X | X | X | X | X | X |
|  | CA3 | X | X | X | X | X | X |
|  | CA4 | X | X | X | X | X | X |
|  | CA5 | X | X | X | X | X | X |
|  | CA6 | X |  |  |  |  |  |
|  | CN1 | X | X | X | X | X | X |
|  | CN2 | X |  |  |  |  |  |
|  | CN3 | X | X | X | X | X | X |
|  | CN4 | X | X | X | X | X | X |
|  | CN5 | X |  |  |  |  |  |
|  | CN6 | X |  |  |  |  |  |
|  | CC1 | X | X | X | X | X | X |
|  | CC2 | X |  |  |  |  |  |
|  | CC3 | X |  |  |  |  |  |
|  | CC4 | X | X | X | X | X | X |
|  | CS1 | X | X | X | X | X | X |
|  | CS2 | X |  |  |  |  |  |
|  | CS3 | X |  |  |  |  |  |
|  | CS4 | X | X | X | X | X | X |
|  | CI1 | X | X | X | X | X | X |
|  | CI2 | X | X | X | X | X | X |
|  | CI3 | X | X | X | X | X | X |
|  | CB1 | X | X | X | X | X | X |
|  | CB2 | X | X | X | X | X | X |
|  | CB3 | X | X | X | X | X | X |
|  | CB4 | X |  |  |  |  |  |
|  | CB5 | X |  |  |  |  |  |
| DI Items | DA1 |  | X |  |  |  |  |
|  | DA2 |  | X |  |  |  |  |
|  | DA3 | X | X | X | X | X | X |
|  | DA4 | X | X | X | X | X | X |
|  | DA5 | X | X | X | X | X | X |
|  | DA6 |  | X |  |  |  |  |
|  | DN1 | X | X | X | X | X | X |
|  | DN2 |  | X |  |  |  |  |
|  | DN3 | X | X | X | X | X | X |
|  | DN4 | X | X | X | X | X | X |
|  | DN5 |  | X |  |  |  |  |
|  | DN6 |  | X |  |  |  |  |
|  | DC1 | X | X | X | X | X | X |
|  | DC2 |  | X |  |  |  |  |
|  | DC3 |  | X |  |  |  |  |
|  | DC4 | X | X | X | X | X | X |
|  | DS1 | X | X | X | X | X | X |
|  | DS2 |  | X | X |  |  |  |
|  | DS3 | X | X | X | X | X | X |
|  | DS4 | X | X | X | X | X | X |
|  | DS5 |  | X | X |  |  |  |
|  | DI1 | X | X | X | X | X | X |
|  | DI2 | X | X | X | X | X | X |
|  | DI3 | X | X | X | X | X | X |
|  | DB1 | X | X | X | X | X | X |
|  | DB2 | X | X | X | X | X | X |
|  | DB3 | X | X | X | X | X | X |
|  | DB4 | X | X | X | X | X | X |
|  | DB5 | X | X | X | X | X | X |
|  | DB6 | X | X | X | X | X | X |
|  | DB7 | X | X | X | X | X | X |
|  | DB8 | X | X | X | X | X | X |
|  | DB9 | X | X | X | X | X | X |
|  | DB10 | X | X | X | X | X | X |
|  | DB11 | X | X | X | X | X | X |
| FA items | FA1 |  |  | X |  |  |  |
|  | FA2 |  |  | X |  |  |  |
|  | FA3 | X | X | X | X | X | X |
|  | FA4 | X | X | X | X | X | X |
|  | FA5 | X | X | X | X | X | X |
|  | FA6 |  |  | X |  |  |  |
|  | FN1 | X | X | X | X | X | X |
|  | FN2 |  |  | X |  |  |  |
|  | FN3 | X | X | X | X | X | X |
|  | FN4 | X | X | X | X | X | X |
|  | FN5 |  |  | X |  |  |  |
|  | FN6 |  |  | X |  |  |  |
|  | FC1 | X | X | X | X | X | X |
|  | FC2 |  |  | X |  |  |  |
|  | FC3 |  |  | X |  |  |  |
|  | FC4 | X | X | X | X | X | X |
|  | FS1 | X | X | X | X | X | X |
|  | FS2 | X | X | X | X | X | X |
|  | FS3 |  |  | X |  |  |  |
|  | FS4 |  |  | X |  |  |  |
|  | FI1 | X | X | X | X | X | X |
|  | FI2 | X | X | X | X | X | X |
|  | FI3 | X | X | X | X | X | X |
|  | FB1 | X | X | X | X | X | X |
|  | FB2 | X | X | X | X | X | X |
|  | FB3 | X | X | X | X | X | X |
|  | FB4 | X | X | X | X | X | X |
|  | FB5 | X | X | X | X | X | X |
|  | FB6 | X | X | X | X | X | X |

Appendix 6: Structural equation models with standardized estimates (and standard errors), controlling for gender, experience, teaching level, and socio-economic level of the school population

| CBP |  | Behavior | Intention | Attitude | Subjective norm | PBC |
| --- | --- | --- | --- | --- | --- | --- |
|  | Teacher gender (ref: Women) | -.020 (.031) | -.049 (.029) | -.119 (.038)** | .048 (.055) | -.047 (.039) |
|  | Teacher experience | -.193 (.032)** | -.089 (.032) | -.175 (.037)** | -.234 (.057)** | -.082 (.039)* |
|  | School level (ref: Cycle 2) |  |  |  |  |  |
|  | Cycle 1 | .091 (.038) | -.010 (.039) | .123 (.046)** | -.037 (.061) | .050 (.049) |
|  | Cycle 3 | -.011 (.037) | -.017 (.034) | .049 (.044) | .012 (.064) | -.026 (.046) |
|  | Cycle 4 | .071 (.037) | -.001 (.034) | .004 (.043) | -.010 (.062) | .007 (.045) |
|  | More than one cycle | .044 (.036) | .010 (.028) | -.008 (.045) | .122 (.070) | -.042 (.045) |
|  | School proportion of socio-culturally  and economically disadvantaged children (ref: Between 40% and 60%) |  |  |  |  |  |
|  | Over 80% | .034 (.033) | .019 (.034) | -.102 (.041)* | -.153 (.062)* | -.087 (.040)* |
|  | Between 60% and 80% | -.042 (.039) | -.047 (.036) | -.047 (.046) | -.119 (.065) | .003 (.048) |
|  | Between 20% and 40% | .027 (.037) | -.008 (.037) | -.012 (.047) | -.133 (.067)* | .063 (.049) |
|  | Less than 20% | -.011 (.041) | -.073 (.038) | .034 (.048) | -.122 (.069) | .065 (.051) |
|  | Attitude | - | .295 (.038)** | - | - | - |
|  | Subjective norm | - | .019 (.044) | - | - | - |
|  | PBC | .338 (.068)** | .587 (.033)** | - | - | - |
|  | Intention | .281 (.070)** | - | - | - | - |
|  | **R^2^** | **43.6%**** | **67.3%**** | **7.7%**** | **9.6%**** | **3.4%**** |
| DI | Teacher gender (ref: Women) | -.059 (.032) | -.127 (.050)* | -.108 (.042)* | .050 (.095) | .016 (.039) |
|  | Teacher experience | -.104 (.030)** | .040 (.051) | -.051 (.041) | -.226 (.087)** | .023 (.038) |
|  | School level (ref: Cycle 2) |  |  |  |  |  |
|  | Cycle 1 | -.017 (.039) | .110 (.070) | .067 (.046) | -.313 (.106)** | -.053 (.044) |
|  | Cycle 3 | -.038 (.037) | .001 (.056) | -.005 (.047) | -.147 (.106) | -.044 (.043) |
|  | Cycle 4 | -.030 (.035) | -.049 (.059) | -.037 (.047) | -.167 (.105) | -.026 (.045) |
|  | More than one cycle | -.027 (.034) | -.059 (.049) | .007 (.045) | .057 (.084) | .001 (.039) |
|  | School proportion of socio-culturally  and economically disadvantaged children (ref: Between 40% and 60%) |  |  |  |  |  |
|  | Over 80% | .037 (.032) | .051 (.052) | -.114 (.041)** | -.012 (.107) | -.081 (.038)* |
|  | Between 60% and 80% | -.039 (.039) | .043 (.053) | -.052 (.047) | -.140 (.097) | .034 (.046) |
|  | Between 20% and 40% | -.001 (.039) | -.030 (.055) | .027 (.048) | .027 (.099) | .081 (.046) |
|  | Less than 20% | -.064 (.039) | .058 (.060) | .002 (.049) | -.216 (.109)* | .055 (.045) |
|  | Attitude | - | .556 (.041)** | - | - | - |
|  | Subjective norm | - | .395 (.096)** | - | - | - |
|  | PBC | .507 (.035)** | .364 (.045)** | - | - | - |
|  | Intention | .068 (.037) | - | - | - | - |
|  | **R^2^** | **34.6%**** | **74.4%**** | **4.5%**** | **21.6%**** | **2.0%*** |
| FA | Teacher gender (ref: Women) | -.069 (.036) | -.053 (.030) | -.037 (.038) | .037 (.066) | -.024 (.040) |
|  | Teacher experience | -.009 (.035) | -.006 (.034) | -.222 (.037)** | -.204 (.063)** | -.107 (.040)** |
|  | School level (ref: Cycle 2) |  |  |  |  |  |
|  | Cycle 1 | -.155 (.042)** | -.060 (.037) | .055 (.047) | -.063 (.070) | -.096 (.047)* |
|  | Cycle 3 | -.058 (.039) | .027 (.036) | .039 (.044) | -.009 (.072) | -.064 (.047) |
|  | Cycle 4 | -.012 (.041) | .014 (.037) | .001 (.045) | .033 (.072) | -.025 (.046) |
|  | More than one cycle | -.070 (.037) | .014 (.038) | .017 (.041) | .034 (.070) | -.022 (.046) |
|  | School proportion of socio-culturally  and economically disadvantaged children (ref: Between 40% and 60%) |  |  |  |  |  |
|  | Over 80% | .010 (.037) | .038 (.032) | -.065 (.039) | -.053 (.069) | -.112 (.040)** |
|  | Between 60% and 80% | -.079 (.041) | -.017 (.041) | -.004 (.048) | -.151 (.078) | -.009 (.050) |
|  | Between 20% and 40% | -.056 (.043) | -.020 (.038) | .049 (.047) | -.104 (.075) | .075 (.050) |
|  | Less than 20% | -.072 (.043) | -.049 (.039) | .064 (.046) | -.036 (.070) | .064 (.050) |
|  | Attitude | - | .354 (.034)** | - | - | - |
|  | Subjective norm | - | .068 (.054) | - | - | - |
|  | PBC | .315 (.067)** | .580 (.035)** | - | - | - |
|  | Intention | .157 (.066)* | - | - | - | - |
|  | **R^2^** | **23.3%**** | **65.3%**** | **6.3%**** | **7.2%*** | **4.6%**** |

Note. * p ≤ .05. ** p ≤ .01
